# An Integrated Metabolomics and Bioactivity Approach to Characterize *Salvia heldreichiana* and *Salvia tomentosa* Extracts

**DOI:** 10.1002/open.70302

**Published:** 2026-09-21

**Authors:** Gokhan Zengin, Nilofar Nilofar, Alina Kalyniukova, Vasil Andruch, Giancarlo Angeles Flores, Paola Angelini, Roberto Venanzoni, Kubra Aslan, Ilhami Gulcin, Evren Yildiztugay

**Affiliations:** ^1^ Deparment of Biology Science Faculty Selcuk University Konya Turkey; ^2^ Botanic Garden “Giardino dei Semplici” Department of Pharmacy “G. d'Annunzio” University Chieti Italy; ^3^ Faculty of Forestry and Wood Sciences Czech University of Life Sciences Prague Praha, Suchdol Czech Republic; ^4^ Department of Analytical Chemistry Institute of Chemistry Faculty of Science P. J. Šafárik University Košice Slovakia; ^5^ Department of Chemistry, Biology and Biotechnology University of Perugia Perugia Italy; ^6^ Centro di Ricerca per l’Innovazione, Digitalizzazione, Valorizzazione e Fruizione del Patrimonio Culturale e Ambientale (CE.D.I.PA.) Spoleto Italy; ^7^ Department of Chemistry Science Faculty Ataturk University Erzurum Turkey; ^8^ Deparment of Biotechnology Science Faculty Selcuk University Konya Turkey

**Keywords:** anti‐diabetic, anti‐microbial, carbonic anhydrase inhibition, metabolomic, natural agents, *Salvia*

## Abstract

The genus *Salvia*, widely known as sage, represents the largest group within the Lamiaceae family and includes numerous species with traditional uses. This study investigates the chemical composition, antioxidant activity, enzyme inhibition, and antibacterial activity of the aerial parts and roots of two *Salvia* species, *Salvia heldreichiana,* and *Salvia tomentosa*. For chemical analysis, ultraperformance liquid chromatography‐quadrupole‐time‐of‐flight mass spectrometry (LC–QTOF–MS) was used. LC–QTOF–MS analysis revealed that the extracts from the aerial parts of *S*. *tomentosa* had a higher number of metabolites than those of *S*. *heldreichiana*, including amino and organic acids, various phenolic compounds, and unknown metabolites. *S*. *heldreichiana* aerial parts water extract possessed the highest antioxidant activity in CUPRAC > FRAP > ABTS > DPPH > MCA > PBD. For the enzyme inhibition, *S*. *heldreichiana* root extracts exhibited the highest anti‐AChE activity, particularly in Ethanol extracts (2.88 mg GALAE/g), while the highest anti‐BChE activity was the ethanol extract of the aerial part (3.65 mg GALAE/g). Consequently, the ethanol/water aerial part extracts, with their superior antioxidant, phenolic, and multienzyme inhibitory profile, present themselves as best candidates of *S*. *heldreichiana* and *S*. *tomentosa* for further in vivo pharmacological evaluation and development.

## Graphical abstract

*S.* *heldreichiana* and *S. tomentosa* can be considered as effective sources of natural bioactive agents for further in vivo pharmacological evaluation and development.

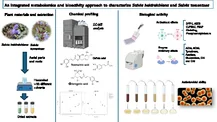

## 1 Introduction

Phytochemicals, also termed phytonutrients, play a vital part in pharmaceuticals in combating chronic disorders by harnessing their abilities in the prevention and treatment of various diseases [1, 2]. Current breakthroughs indicate that approximately 80% of the global population expects the benefits offered by medications and drugs from such chemical sources, thereby affecting pharmaceuticals [3]. Of the prescription medications in the United States, nearly 25% of them are comprised of phytochemicals [4]. Around 80,000 species of plants are utilized as various forms of phytochemical medications due to their demonstrated effectiveness in combating both chronic and acute conditions, as reported by the WHO, as their side effects are less toxic compared to other forms of chemical medications [5]. In the last century, 121 medicinal medications consisting of phytochemicals were discovered globally, most of which existed in ancient systems of knowledge dating back to 2800 BCE [5].

Plants are also known to be rich in bioactive natural compounds, such as low‐molecular‐weight secondary plant metabolites and antioxidants [6, 7]. However, the predominant class of these bioactive compounds is the phenolic compounds. These are involved in several vital functions within the plant, including plant growth, coloration, reproduction, pathogen protection, and herb protection. This is primarily owing to the phytoalexin property as these are highly astringent in nature [8, 9]. These exhibit various bioactivities, such as antioxidant, anti‐inflammatory, anti‐allergic, anti‐viral, hepatoprotective, and anticarcinogenic properties, in plants. However, the antioxidant property has gained considerable attention, especially owing to the reduction in the production of free radicals [10, 11, 12].

*Salvia*, the largest genus of Lamiaceae, includes about 900 species, which are geographically widespread, especially in Europe and North America, where the plant has been cultivated as well as naturalized [13, 14]. There are 107 species of the *Salvia* plant in Turkey, out of which 58 are endemic, and the plant is commonly known as “shalba” or “adacayi” [15]. Different *Salvia* species have economic and medicinal value owing to their essential oils and phenolic compounds with antioxidant, antimicrobial, and anti‐inflammatory properties [16, 17, 18, 19]. In addition, these biological compounds also inhibit lipoperoxidation and have been used in the preservation of fat‐containing products [20, 21, 22]. *Salvia officinalis*, also known as sage, has long recognized as a culinary herb and in folk medicine for its antiseptic, astringent, and spasmolytic actions and is one of the famous species of this genus [22, 23]. Various regions, including Asia, Europe, and Latin America, have utilized *Salvia* species to manage conditions such as inflammation, ulcers, seizures, digestive troubles, high blood sugar, and intellectual impairments [16, 24, 25].

*Salvia heldreichiana* Boiss. Ex Bentham is an endemic plant of Turkey, and it is known as “ayaklı şalba” [26]. Its essential oil has been reported to be rich in α‐pinene, linalool, and β‐pinene [27, 28]. In an earlier study by Erdogan et al. [29], the essential oil of *S. heldreichiana* was found to contain a high proportion of oxygenated sesquiterpenes (78.9%) and showed cytotoxic activity against the A375, PC‐3, and MCF‐7 cell lines. In addition, Erdogan et al. [30] reported that the essential oil of *S. heldreichiana* displayed a good antimicrobial activity against the tested bacteria and fungal strains. The methanol extract of *S. heldreichiana* was investigated for antioxidant and enzyme inhibitory properties by Yilmaz et al. [31], and the extract exhibited significant ability. Furthermore, in that same study, rosmarinic acid was determined to be the main constituent of the tested extract. In a recent study by Dastan et al. [28], the essential oil of *S. heldreichiana* exhibited rapid‐onset anxiolytic effect.

*Salvia tomentosa* Mill., an annual or perennial herb that is widely distributed in Anatolia, has tomentose stems and Purple‐Lilac to White Corollas. Its aerial parts have been used in traditional medicine as an herbal tea for treating rheumatic, abdominal, and stomach pain. It has been reported that an essential oil of *S. tomentosa* has been demonstrated to be effective as an antimicrobial agent, while an aqueous methanol solution of *S. tomentosa* has been evident as an antioxidizing agent. In another study, the dichloromethane extracts of the plant demonstrate cytotoxic effects against different cancer cell lines [15, 32, 33]. In a recent study by Karahan et al. [34], silver nanoparticles were synthesized using the leaf extract of *S. tomentosa*, and these nanoparticles showed promising antimicrobial activity for the development of functional applications. Kayicilar and Maral [26] found that α‐pinene was the main component in the essential oil of *S. tomentosa*. In a recent investigation conducted by Saltan et al. [35], the ethanolic extract of S. tomentosa exhibited the minimal cytotoxic effect on NIH/3T3 cells, a noncancerous cell line.

To date, phytochemical work on these two species has been largely confined to their essential oils or to a single aerial‐part extract examined in isolation with a limited, targeted assay panel, while the roots, the influence of extraction solvent, and several pharmacologically relevant enzyme targets have remained unexplored. To the best of our knowledge, this is the first study to combine untargeted LC–QTOF–MS metabolomic profiling of both the aerial parts and roots of *S. heldreichiana* and *S. tomentosa*, obtained with different solvents, with a multiassay antioxidant battery (CUPRAC, FRAP, ABTS, DPPH, metal‐chelating, and phosphomolybdenum assays) and a broad enzyme‐inhibition panel covering cholinesterases (AChE, BChE), carbohydrate‐hydrolyzing enzymes (α‐amylase, α‐glucosidase), carbonic anhydrase isoenzymes I and II (hCA I and hCA II) and tyrosinase, together with antimicrobial activity. This integrated metabolomics‐bioactivity approach makes it possible to link organ‐ and solvent‐specific chemical fingerprints directly to biological activity and to pinpoint the most promising extracts of these two Turkish *Salvia* species for further pharmacological development, a level of comparative, multiorgan, and multitarget characterization that has not been previously reported.

## 2 Materials and Methods

### 2.1 Plant Collection

Plant specimens were collected in 2022 from Turkey (*S. tomentosa*: Kestel, Konya, 1290 m; *S. helderichiana*: between Mut and Karaman, 20. Km, 1350 m). Dr. Evren Yildiztugay carried out the formal botanical classification of the material. A voucher specimen (EY‐3163 for *S. tomentosa* and EY‐3153 for *S. heldreichiana*) was placed in the Faculty of Science at Selçuk University. Approximately twenty individual plants were sampled from a single population. Plant material was first rinsed under tap water to remove soil and other debris and then given a final rinse with distilled water. Aerial parts and roots were separated by hand and left to dry in a shaded, well‐ventilated room at the Department of Biology, Selçuk University, for around ten days. Once dry, the material was ground into powder using a laboratory mill (Retsch, SM‐200) and extracted the same week. Between grinding and extraction, the powdered samples were kept in the dark under well‐ventilated conditions at roughly 20 °C. All chemical analyses were carried out about 1 month after the plants had been collected.

### 2.2 Plant Extract Preparation

Four solvent systems were used to obtain bioactive constituents: ethyl acetate, ethanol, ethanol–water (70%), and distilled water. For each extraction, 10 g of the prepared plant material was mixed with 200 mL of the corresponding solvent. The methodology was adapted according to the properties of the solvent. Extractions using organic solvents were performed via maceration for 24 h under ambient conditions. In contrast, the water‐based extraction utilized a 15 min infusion with heated water. Following extraction, the products obtained were concentrated using distinct techniques. The aqueous extract was stabilized by lyophilization, while the organic solvent fractions were recovered by evaporating the solvents under reduced pressure using a rotary evaporator.

### 2.3 Total Phenolic and Flavonoid Contents

Total phenolic and flavonoid contents of the extracts were determined using established colorimetric assays, following the referenced procedure [36].

In the total phenolic content (TPC) assay, diluted Folin–Ciocalteu reagent (1 mL, 1:9, v/v) was combined with sample solution (0.25 mL) and vigorously shaken. Na_2_CO_3_ solution (0.75 mL, 1%) was added after 3 min, and the sample absorbance was measured at 760 nm after 2 h of room‐temperature incubation. Milligrams of gallic acid equivalents (mg GAE/g extract) were used to express the TPC.

The AlCl_3_ technique was used to calculate the total flavonoid content. In short, the same volume of aluminum trichloride (2%) in methanol was combined with the sample solution (1 mL). In a similar manner, 1 mL of sample solution was added to 1 mL of methanol without AlCl_3_ to create a blank. Following a 10 min incubation period at room temperature, the sample and blank absorbances were measured at 415 nm. The sample's absorbance was deducted from the blank's. The total flavonoid content was measured in milligrams of rutin equivalents (mg RE/g extract) using rutin as a reference standard.

### 2.4 Antioxidant Assays

Antioxidant capacity was assessed using a panel of in vitro assays following a previously described protocol [37]. Reducing power was evaluated using FRAP (ferric‐reducing antioxidant power) and CUPRAC (cupric‐reducing antioxidant capacity), while radical scavenging was measured using DPPH (2,2‐diphenyl‐1‐picrylhydrazyl) and ABTS (2,2′‐azino‐bis(3‐ethylbenzothiazoline‐6‐sulfonic acid)) assays. Results from these four assays were quantified and standardized to Trolox and reported as mg Trolox equivalents (TE) per g of dried extract (mg TE/g). Total antioxidant capacity was additionally determined by the phosphomolybdenum method (PBD assay) and expressed as mmol Trolox equivalents per g (mmol TE/g). Finally, metal‐chelating activity was evaluated using a chelation assay and reported as mg EDTA equivalents (EDTAE) per g of extract (mg EDTAE/g).

### 2.5 Enzyme Inhibitory Activity Assays

The enzyme inhibitory potential of the extracts was evaluated against five key targets; acetylcholinesterase (AChE), butyrylcholinesterase (BChE), tyrosinase, α‐amylase, and α‐glucosidase; using established colorimetric procedures [37]. To standardize the results, inhibition was quantified using established reference compounds. Cholinesterase inhibitory activity was expressed as mg galanthamine equivalents (GALAE) per g of extract (mg GALAE/g). α‐Amylase and α‐glucosidase inhibitory activities were expressed as mg acarbose equivalents (ACAE) per g (mg ACAE/g), while tyrosinase inhibition was reported as mg kojic acid equivalents (KAE) per g (mg KAE/g). In addition, the extracts were evaluated for inhibitory activity against human carbonic anhydrase isoenzymes I and II (hCA I and hCA II). Human carbonic anhydrase enzyme was purified by Sepharose 4B‐L‐tyrosine sulfanilamide affinity column and characterized by SDS‐PAGE as previously applied. The esterase activity of the carbonic anhydrase (CA) enzyme yields a yellow‐colored aromatic p‐nitrophenolate compound with pH around pH = 7.5. By taking advantage of this transformation, the esterase activity of CA enzymes can be determined spectrophotometrically [38]. Various concentrations of the samples in 0.05 M pH = 7.4 Tris‐SO_4_, 0.07 mM of p‐nitrophenylacetate (in 1:25 acetone:water), and 20 µL of the enzyme were gently vortexed, and as soon as the addition of the enzyme, the absorbance change at 348 nm was monitored by measuring minute intervals for 3 min. Control reactions and blank reactions were set up without inhibitors and enzymes. These steps were repeated until more than half of enzyme activity was inhibited.

### 2.6 LC–MS Metabolomic Analysis

Metabolomic analysis was performed using an Agilent 1290 Infinity II LC system coupled to an Agilent 6546 quadrupole time‐of‐flight mass spectrometer (QTOF–MS; Agilent Technologies, USA). Chromatographic separation was achieved on an Agilent InfinityLab Poroshell 120 EC‐C18 column (2.1 × 150 mm and 2.7 µm particle size). The mobile phases consisted of water with 0.1% formic acid (solvent A) and methanol (solvent B). The gradient elution was programmed as follows: 0–4 min, 85% A; 4–7 min, 75% A; 7–9 min, 68% A; 9–16 min, 60% A; 16–22 min, 45% A; and 22–28 min, 5% A, followed by a 2 min hold at 5% A. The flow rate was 0.5 mL/min, the column temperature was maintained at 35 °C, and the injection volume was 1 µL.

Mass spectrometric data were acquired in both positive and negative electrospray ionization modes. The QTOF settings included a scan range of *m*/*z* 100–1000, drying gas temperature of 160 °C, sheath gas flow rate of 12.0 L/min, sheath gas temperature of 400 °C, capillary voltage of 5.0 kV, nozzle voltage of 2.0 kV, fragmentor voltage of 140 V, and collision energies of 10, 20, and 40 eV. MS/MS spectra were acquired in a scan range of *m*/*z* 50–800 using a retention time window of 0.5 min, an isolation window of 1.3 amu, and a spectral acquisition rate of 3 spectra per second. Continuous mass correction was enabled using reference masses at *m*/*z* 112.9855 and 966.0007.

Data processing, including feature extraction and chromatographic alignment, was carried out using an Agilent MassHunter Profinder 10.0. Features were extracted within an *m*/*z* range of 100–1000 using a mass accuracy tolerance of 2 mDa and a retention time window of ±0.25 min. Chromatographic alignment was performed with a maximum retention time shift of 0.5 min, and only features with a minimum intensity of 5000 counts were retained for further analysis. A pooled quality control (QC) sample was prepared by combining 100 μL of each extract and was analyzed throughout the analytical sequence to monitor instrument stability and data quality. Pure methanol was used as a blank, and all features detected in the blank samples were removed from the dataset prior to statistical analysis. To improve data quality, only features detected in at least three samples within one experimental group and present in the QC sample were retained for further analysis. Missing values were replaced with the minimum intensity threshold (5000 counts).

The resulting feature matrix, comprising all extraction solvents and plant organs, was imported into MetaboAnalyst 6.0 for statistical analysis (https://www.metaboanalyst.ca/). Peak intensities were median normalized across samples and log_10_ transformed to reduce skewness and scaling by mean centering before multivariate analysis.

Principal component analysis (PCA) was used as an unsupervised approach to visualize similarities and differences among the samples. The statistical significance of sample grouping was evaluated using PERMANOVA with 999 permutations. Partial least squares‐discriminant analysis (PLS‐DA) was applied for sample classification, and variable importance in projection (VIP) scores >1.0 were calculated to identify discriminative features. Hierarchical clustering analysis was performed using Euclidean distance and Ward's linkage method. Heatmaps were generated from metabolites showing significant differences among extraction solvents based on one‐way ANOVA (*p* ≤ 0.05).

Metabolites were identified by MS/MS analysis through comparison with the METLIN database, an in‐house spectral library, and published literature, considering accurate mass, retention time, isotope distribution, and characteristic fragmentation patterns. The confidence of metabolite identification was assigned according to the recommendations of the Metabolomics Standards Initiative (MSI). Metabolites. Metabolites confirmed by comparison with reference standards were classified as MSI Level 1. Metabolites identified based on accurate mass, MS/MS fragmentation, and database and literature matching, but without confirmation using authentic reference standards, were classified as putatively annotated compounds (MSI Level 2). Features for which only the compound class could be assigned based on their fragmentation characteristics were classified as tentatively characterized compounds (MSI Level 3). Unknown features belonged to MSI Level 4.

### 2.7 Antimicrobial Activity Assays

The antimicrobial activity of *Salvia tomentosa* and S. heldreichiana was evaluated against 17 microbial isolates, including bacteria, yeasts, and dermatophytes, using CLSI‐based minimum inhibitory concentration (MIC) assays. The yeast panel comprised *Candida tropicalis* (YEPGA 6184), *C. albicans* (YEPGA 6379), and *C. parapsilosis* (YEPG 6551).

The bacterial panel included Gram‐strains—*Escherichia coli* (ATCC 10536), *Pseudomonas aeruginosa* (ATCC 15442), and *Salmonella typhi* (ATCC 15442)—and Gram + strains—*Bacillus cereus* (ATCC 12826), *B. subtilis* (PeruMycA 6), and *Staphylococcus aureus* (ATCC 6538).

The dermatophyte collection consisted of *Trichophyton mentagrophytes* (CCF 4823), *T. tonsurans* (CCF 4834), *T. rubrum* (CCF 4933), *Arthroderma quadrifidum* (CCF 5792), *T. erinacei* (CCF 5930), *A. gypseum* (CCF 6261), *A. currey* (CCF 5207), and *A. insingulare* (CCF 5417).

QC for antifungal susceptibility testing was performed using *Candida parapsilosis* ATCC 22019 and *C. krusei* ATCC 6258, in accordance with CLSI documents M27‐A3, M38‐A2, M27‐S4, and Supplement M61 [39, 40, 41, 42].

All reference strains are preserved in the PeruMycA culture collection (Department of Chemistry, Biology, and Biotechnology, University of Perugia, Italy) and are available upon request.

### 2.8 Antibacterial Susceptibility Testing

Antibacterial activity was assessed by determining MICs using the broth dilution method, following CLSI guideline M07‐A9. Bacterial inocula were obtained from 24 h cultures grown on tryptic soy agar and suspended in Mueller–Hinton broth (MHB). After overnight incubation, suspensions were adjusted to approximately 1–2 × 10^8^ CFU/mL (0.5 McFarland standard) and further diluted in MHB to achieve a final inoculum of 5 × 10^5^ CFU/mL in each test tube. Inoculum accuracy and reproducibility were verified by serial dilution and plating on Mueller–Hinton agar.

Each assay included growth controls (MHB with inoculum but without extract), sterility controls (uninoculated MHB containing extract), and QC strains (*Escherichia coli* ATCC 25922 and *Staphylococcus aureus* ATCC 29213), tested in parallel to confirm assay performance and compliance with CLSI reference MIC ranges. MICs were read after incubation at 35 °C for 18–20 h under aerobic conditions and defined as the lowest extract concentration that completely inhibited visible bacterial growth [43].

### 2.9 Antifungal Susceptibility Testing

Susceptibility testing of yeasts and dermatophytes was performed according to CLSI protocols [39, 40, 41, 42, 44]. Assays were conducted in RPMI 1640 medium supplemented with 2% (w/v) glucose and L‐glutamine and buffered to pH 7.0 with 0.165 mol/L MOPS.

Fungal isolates were cultured on Sabouraud dextrose agar at 25 °C for 7 days. Inocula were standardized spectrophotometrically (OD600 = 0.09–0.11) and diluted 1:50 in RPMI 1640 to obtain final concentrations of 0.2–0.4 × 10^4^–10^5^ CFU/mL. Inoculum density was verified by plating serial dilutions on Sabouraud dextrose agar.

MICs (µg/mL) were recorded after incubation at 30 °C under ambient air for 24 h (yeasts) or 72 h (dermatophytes). Antifungal activity is reported as geometric mean MICs and ranges derived from three independent biological replicates.

### 2.10 Statistical Analysis

All experiments were conducted in triplicate (technical replications), and differences among extracts were evaluated for statistical significance. One‐way ANOVA was performed in GraphPad Prism (version 9.2), followed by Tukey's post hoc test for pairwise comparisons. Statistical significance was set at *p* < 0.05.

## 3 Results and Discussion

### 3.1 Total Phenolic and Flavonoid Content

Phenolic and flavonoids are widely distributed secondary metabolites of the plants known for their antioxidant activity and diverse health benefits [45]. *S*. *heldreichiana* has been extensively investigated by numerous researchers for its essential oil composition [29, 46, 47], whereas only a limited number of studies have reported its total phenolic and flavonoid contents [48]. Therefore, the current study investigates the total phenolic content (TPC) and total flavonoid content (TFC) using various extraction solvents and both the aerial and root parts of *S*. *heldreichiana*. In the current study, the TPC and TFC exhibited distinct variations across *Salvia* species, plant parts, and solvent extracts. The aerial parts of both plants exhibited the highest TPC and TFC contents, as given in Table 1. For *S. heldreichiana*, the aerial part water extracts contained the highest TPC, recorded at 94.36 mg GAE/g, followed by the root water extracts, which recorded 92.81 mg GAE/g. TFC for aerial part was highest in the ethanol extract, followed by water extract, measuring 29.70 and 24.00 mg RE/g. In contracts, the EA extract of both parts of *S. heldreichiana* showed lower contents of TPC and TFC. This trend partially agrees with previous reports [6], in which EA fractions of *S. heldreichiana* were enriched in flavonoids despite noticeable variations in TPC across different solvents.

**TABLE 1:** Extraction yields (%), total phenolic and flavonoid content in the tested extracts.

*Salvia* species	Parts	Extracts	Yields, %	TPC, mg GAE/g	TFC, mg RE/g
*S. heldreichiana*	Aerial parts	Ethyl acetate	1.53	36.79 ± 1.12^h^	9.75 ± 0.19^g^
		Ethanol	2.38	68.90 ± 0.47^f^	16.31 ± 1.27^d^
		Ethanol/water	4.54	90.92 ± 0.43^b^	29.70 ± 0.75^a^
		Water	3.34	94.36 ± 1.40^a^	24.00 ± 0.54^b^
	Roots	Ethyl acetate	0.56	37.19 ± 0.70^h^	4.88 ± 0.12^hi^
		Ethanol	0.82	82.75 ± 0.54^d^	12.76 ± 0.40^ef^
		Ethanol/water	2.43	91.77 ± 1.30^b^	10.76 ± 0.36^g^
		Water	1.95	92.81 ± 0.70^ab^	12.99 ± 0.19^ef^
*S. tomentosa*	Aerial parts	Ethyl acetate	3.69	27.72 ± 0.50^i^	6.09 ± 0.30^hi^
		Ethanol	5.18	68.31 ± 0.63^f^	29.19 ± 1.04^a^
		Ethanol/water	11.75	87.77 ± 0.88^c^	20.54 ± 0.30^c^
		Water	7.08	91.07 ± 0.61^b^	14.08 ± 0.27^e^
	Roots	Ethyl acetate	0.88	26.13 ± 0.16^i^	4.18 ± 0.17^i^
		Ethanol	2.13	64.69 ± 0.35^g^	11.01 ± 1.60^fg^
		Ethanol/water	3.65	78.53 ± 1.09^e^	4.88 ± 0.17^hi^
		Water	3.22	88.21 ± 0.80^c^	6.77 ± 0.39^h^

*Note*: Values are reported as mean ± SD of three parallel measurements. Different letters indicate significant differences between the tested extracts (*p* < 0.05).

Abbreviations: GAE, gallic acid equivalent; RE, rutin equivalent.

In *S. tomentosa*, the aerial part water extracts showed the highest TPC, measuring 91.07 mg GAE/g, followed by the root water extract, which measured 88.21 mg GAE/g. In contrast, the EA extracts of both parts of the plants showed lower values. The highest amount of TFC was found in the aerial parts in the ethanol extract (29.19 mg RE/g), followed by the ethanol/water extracts (20.54 mg RE/g). Consistent with our findings, Ulubelen et al. [49] reported that the ethanol extract of *S*. *tomentosa* was particularly rich in flavonoid constituents, highlighting ethanol as an effective solvent for flavonoid extraction in *Salvia* species.

When the results were evaluated collectively, substantial differences in TPC and TFC were found between the two plant species depending on the solvents used. Water can effectively extract phenolic compounds because the hydroxyl groups of many phenolics form hydrogen bonds with water, while water simultaneously penetrates the plant tissue, hydrates and swells the cellulose–hemicellulose–pectin network, increases membrane permeability, and facilitates the release and diffusion of intracellular and cell‐wall‐associated phenolics into the solvent [50]. Its efficiency is particularly high for polar compounds such as phenolic acids, flavonoid glycosides, catechins, and tannins, although it is generally less effective for poorly polar flavonoid aglycones. Increasing the extraction temperature lowers water viscosity and surface tension, improves tissue wetting and solvent penetration, accelerates molecular diffusion, and softens the cell‐wall matrix; under pressurized subcritical conditions, water also becomes less polar and can dissolve moderately hydrophobic phenolics. Therefore, optimized hot‐water extraction can provide an efficient, safe, economical, and environmentally friendly approach for recovering phenolic compounds [51].

Certain solvents preferentially extract phenolics (e.g., water extract); in contrast, others were more efficient for flavonoids (notably ethanol/water extract), highlighting the importance of solvent selection when targeting specific classes of bioactive compounds within the *Salvia* genus. In agreement with our results showing high TPC in water extracts, our previous findings indicate a high TPC in water extracts in various other *Salvia* species than in alcoholic extracts [52, 53], indicating that water is an effective solvent for the extraction of polyphenolics. Taken together, these results suggest that solvent polarity, rather than plant part alone, is the main determinant of phenolic and flavonoid yield in both species, a pattern that is examined further below in relation to their antioxidant and enzyme‐inhibitory profiles.

### 3.2 Chemical Composition

To study the metabolic diversity between *S. heldreichiana* and *S. tomentosa* LC–MS metabolomic profiling was conducted on extracts from aerial parts and roots using four solvents: ethyl acetate, ethanol, ethanol/water, and water. Data were acquired separately in negative and positive electrospray ionization modes. In total, 207 molecular features were detected, and 88 were identified in negative ionization mode and 171 and 46 in positive ionization mode, respectively.

**FIGURE 1:**
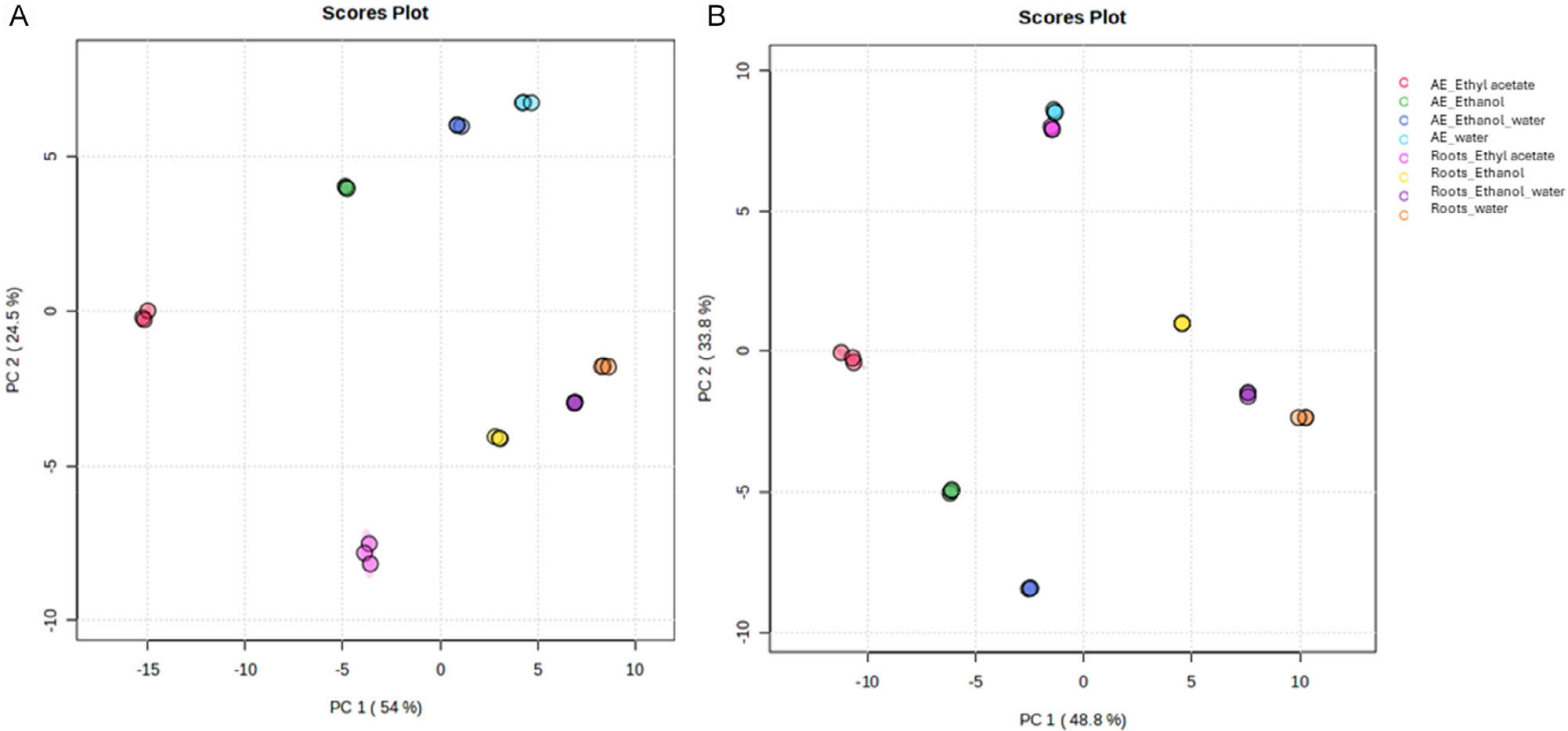
PCA analysis of *Salvia tomentosa* extracts from aerial parts (AE) and roots in negative (A) and positive (B) ionization mode.

**FIGURE 2:**
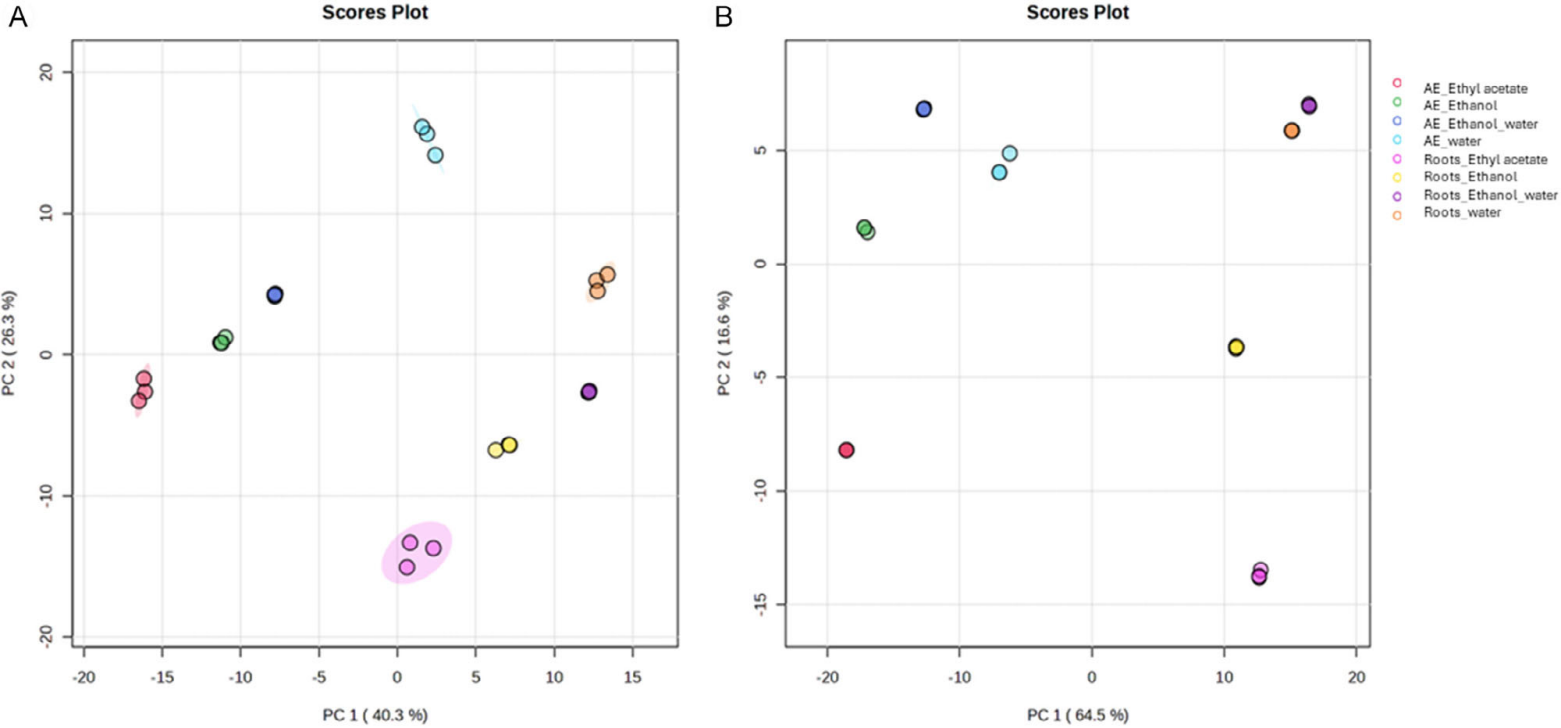
PCA analysis of *Salvia heldreichiana* extracts from aerial parts (AE) and roots in negative (A) and positive (B) ionization mode.

In *S. heldreichiana*, PCA obtained from negative ionization mode (Figure 1A) revealed a clear separation of samples according to both plant part and solvent, with the first two principal components explaining 66.6% of the total variance (PC1: 40.3% and PC2: 26.3%). Extracts clustered distinctly based on solvent type, indicating a strong solvent influence on metabolite composition. Then, samples were separated by plant part to find difference between aerial and root parts. For example, EA extracts from the aerial parts were clearly separated from those from the roots. These patterns were statistically confirmed by PERMANOVA (*F* = 1033, *R* ^2^ = 0.99779, and *p* = 0.001). In the positive ionization mode (Figure 2B), PCA showed clear separation, explaining 81.1% of the variance (PC1: 64.5% and PC2: 16.6%). The extracts grouped according to extraction solvent and plant parts, with distinct compositional profiles. Statistical evaluation confirmed the significance of these differences (*F* = 13,562, *R* ^2^ = 0.99983, and *p* = 0.0010). Similarly, *S. tomentosa* showed pronounced metabolic variability across both plant parts and extraction solvents. In the positive ionization mode (Figure 2B), PCA accounted for 82.6% of the total variance (PC1: 48.8% and PC2: 33.8%). Aerial and root extracts formed separate clusters. EA and ethanol/water extracts were clearly separated from water and ethanol extracts from the aerial part, while root extracts clustered independently. These differences were highly significant (*F* = 10,742, *R* ^2^ = 0.99979, and *p* = 0.001). In negative ionization mode (Figure 2A), *S. tomentosa* samples also showed strong separation, with PC1 and PC2 explaining 54.0% and 24.5% of the variance, respectively, with the total variation of 78.5%. Root extract obtained with ethyl acetate, formed the most distinct cluster that confirmed the solvent selectivity for extraction of specific metabolites. The results were confirmed statistically (*F* = 6612.8, *R* ^2^ = 0.99965, and *p* = 0.001).

**FIGURE 3:**
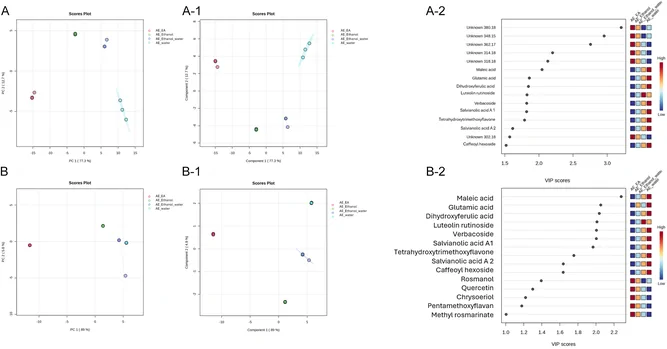
PCA (A) (PERMANOVA: *F*‐value: 646.84; *R* ^2^: 0.99589; *p*‐value (based on 999 permutations): 0.001), PLS‐DA (A‐1) (*R* ^2^ = 0.9998, *Q* ^2^_cum_ = 0.9952, permutation test *p * =  0.001) analysis and 15 important features (VIP  >  1) (A‐2) obtained from all detected features of *Salvia tomentosa* extracts from aerial parts; PCA (B) (*F*‐value: 93.687; *R* ^2^: 0.97232; *p*‐value (based on 999 permutations): 0.002), PLS‐DA (B‐1) (*R* ^2^ = 0.9997, *Q* ^2^_cum_ = 0.9830, permutation test *p * =  0.001) analysis and 15 important features (VIP > 1) (B‐2) obtained from identified compounds of *Salvia heldreichiana* extracts from aerial parts (AE) in negative ionization mode.

**FIGURE 4:**
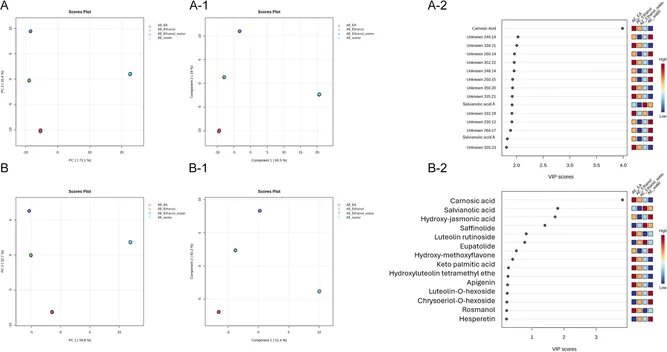
PCA (A) (PERMANOVA: *F*‐value: 116,230; *R* ^2^: 0.99998; *p*‐value (based on 999 permutations): 0.001), PLS‐DA (A‐1) (*R* ^2^ = 0.9999, *Q* ^2^_cum_ = 0.9998, permutation test *p * =  0.001) analysis and 15 important features (VIP  > 1) (A‐2) obtained from all detected features of *Salvia tomentosa* extracts from aerial parts; PCA (B) (*F*‐value: 192,660; *R* ^2^: 0.99999; *p*‐value (based on 999 permutations): 0.001), PLS‐DA (B‐1) (*R* ^2^ = 0.9999, *Q* ^2^_cum_ = 0.9999, permutation test *p * =  0.001) analysis and 15 important features (VIP  >  1) (B‐2) obtained from identified compounds of *Salvia heldreichiana* extracts from aerial parts (AE) in positive ionization mode.

**FIGURE 5:**
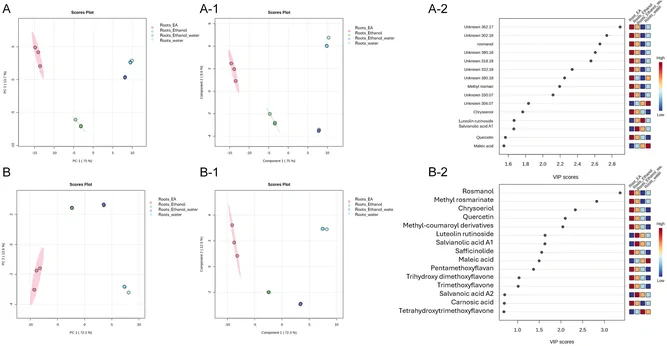
PCA (A) (PERMANOVA: *F*‐value: 487.58; *R* ^2^: 0.99456; *p*‐value (based on 999 permutations): 0.001), PLS‐DA (A‐1) (*R* ^2^ = 0.9989, *Q* ^2^_cum_ = 0.9968, permutation test *p * =  0.001) analysis and 15 important features (VIP  > 1) (A‐2) obtained from all detected features of *Salvia tomentosa* extracts from roots; PCA (B) (*F*‐value: 633.6; *R* ^2^: 0.99581; *p*‐value (based on 999 permutations): 0.001), PLS‐DA (B‐1) (*R* ^2^ = 0.9999, *Q* ^2^_cum_ = 0.9999, permutation test *p * =  0.001) analysis and 15 important features (VIP > 1) (B‐2) obtained from identified compounds of *Salvia heldreichiana* extracts from aerial parts (AE) in negative ionization mode.

**FIGURE 6:**
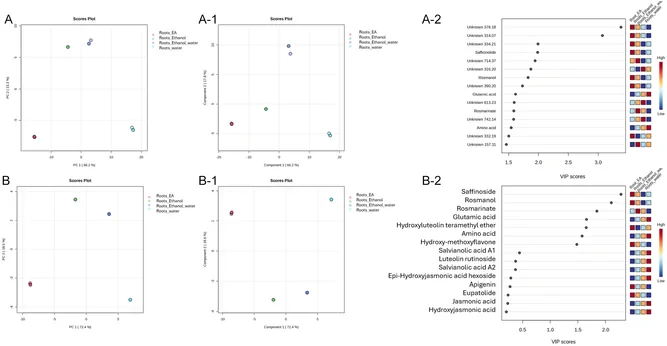
PCA (A) (PERMANOVA: *F*‐value: 9114.7; *R* ^2^: 0.99971; *p*‐value (based on 999 permutations): 0.001), PLS‐DA (A‐1) (*R* ^2^ = 0.9999, *Q* ^2^_cum_ = 0.9999, permutation test *p * =  0.001) analysis and 15 important features (VIP  >  1) (A‐2) obtained from all detected features of *Salvia tomentosa* extracts from roots; PCA (B) (*F*‐value: 225,910; *R* ^2^: 0.99999; *p*‐value (based on 999 permutations): 0.001), PLS‐DA (B‐1) (*R* ^2^ = 0.9999, *Q* ^2^_cum_ = 0.9999, permutation test *p * =  0.001) analysis and 15 important features (VIP  >  1) (B‐2) obtained from identified compounds of *Salvia heldreichiana* extracts from aerial parts (AE) in positive ionization mode.

**FIGURE 7:**
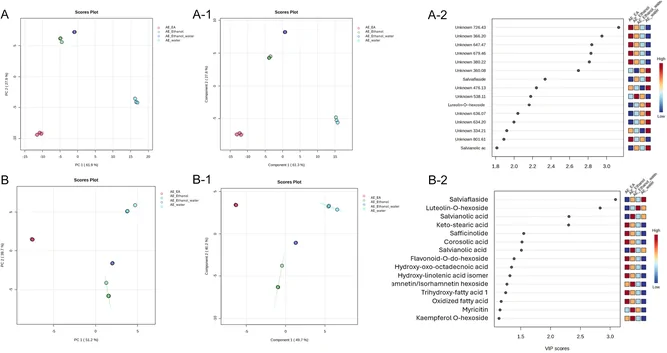
PCA (A) (PERMANOVA: *F*‐value: 3020; *R* ^2^: 0.99912; *p*‐value (based on 999 permutations): 0.001), PLS‐DA (A‐1) (*R* ^2^ = 0.9985, *Q* ^2^_cum_ = 0.9961, permutation test *p * =  0.001) analysis and 15 important features (VIP  >  1) (A‐2) obtained from all detected features of *Salvia heldreichiana* extracts from **aerial parts**; PCA (B) (*F*‐value: 389.49; *R* ^2^: 0.9932; *p*‐value (based on 999 permutations): 0.001), PLS‐DA (B‐1) (*R* ^2^ = 0.9924, *Q* ^2^_cum_ = 0.9824, permutation test *p * =  0.001) analysis and 15 important features (VIP  >  1) (B‐2) obtained from identified compounds of *Salvia heldreichiana* extracts from aerial parts (AE) in negative ionization mode.

**FIGURE 8:**
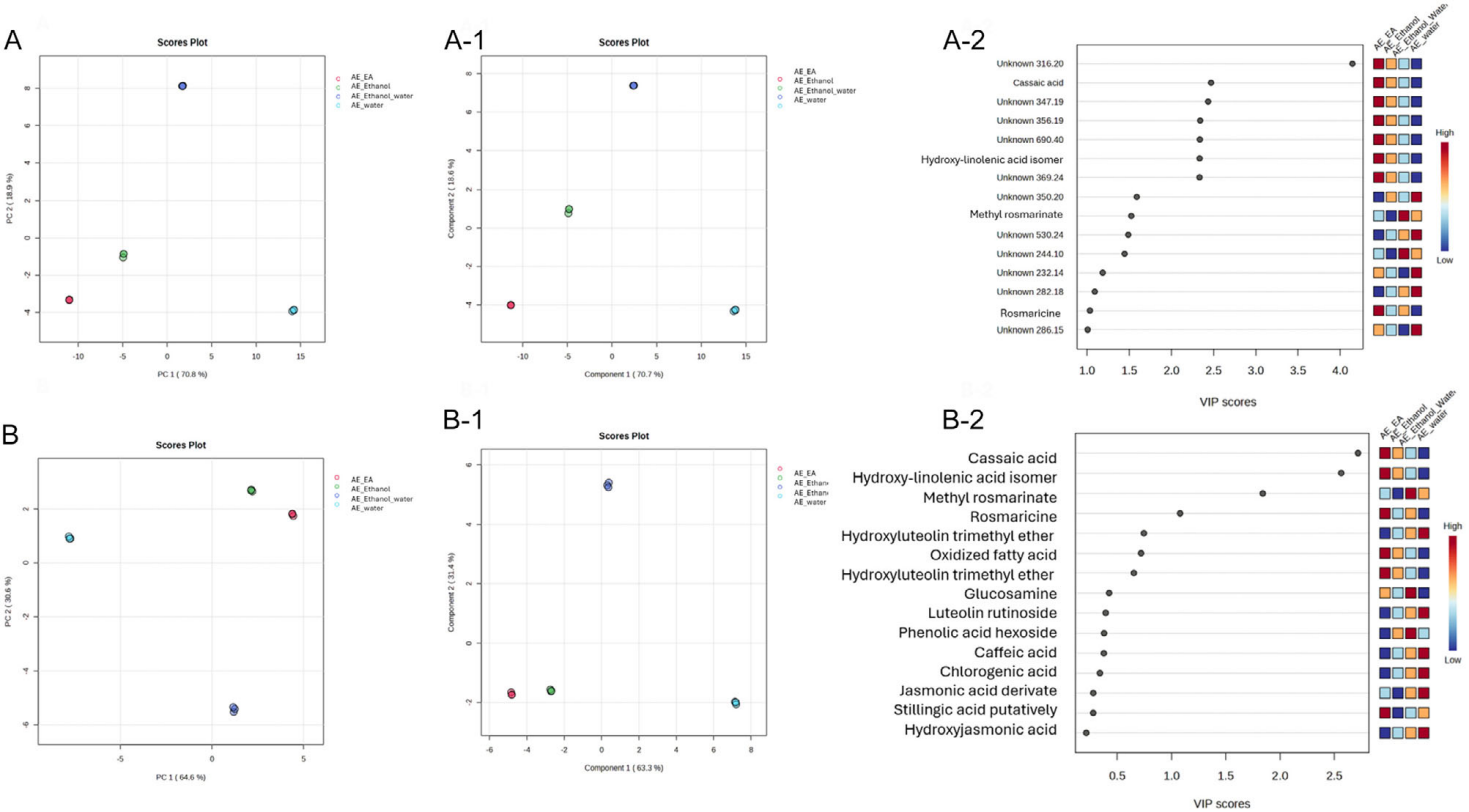
PCA (A) (*F*‐value: 62,138; *R* ^2^: 0.99996; *p*‐value (based on 999 permutations): 0.001), PLS‐DA (A‐1) (*R* ^2^ = 0.9985, *Q* ^2^_cum_ = 0.9961, permutation test *p * =  0.001) analysis and 15 important features (VIP  > 1) (A‐2) obtained from all detected features of *Salvia heldreichiana* extracts from **aerial parts**; PCA (B) (*F*‐value: 26,944; *R* ^2^: 0.9999; *p*‐value (based on 999 permutations): 0.001), PLS‐DA (B‐1) (*R* ^2^ = 0.9999, *Q* ^2^_cum_ = 0.9998, permutation test *p * =  0.001) analysis and 15 important features (VIP  >  1) (B‐2) obtained from identified compounds of *Salvia heldreichiana* extracts from aerial parts (AE) in positive mode.

**FIGURE 9:**
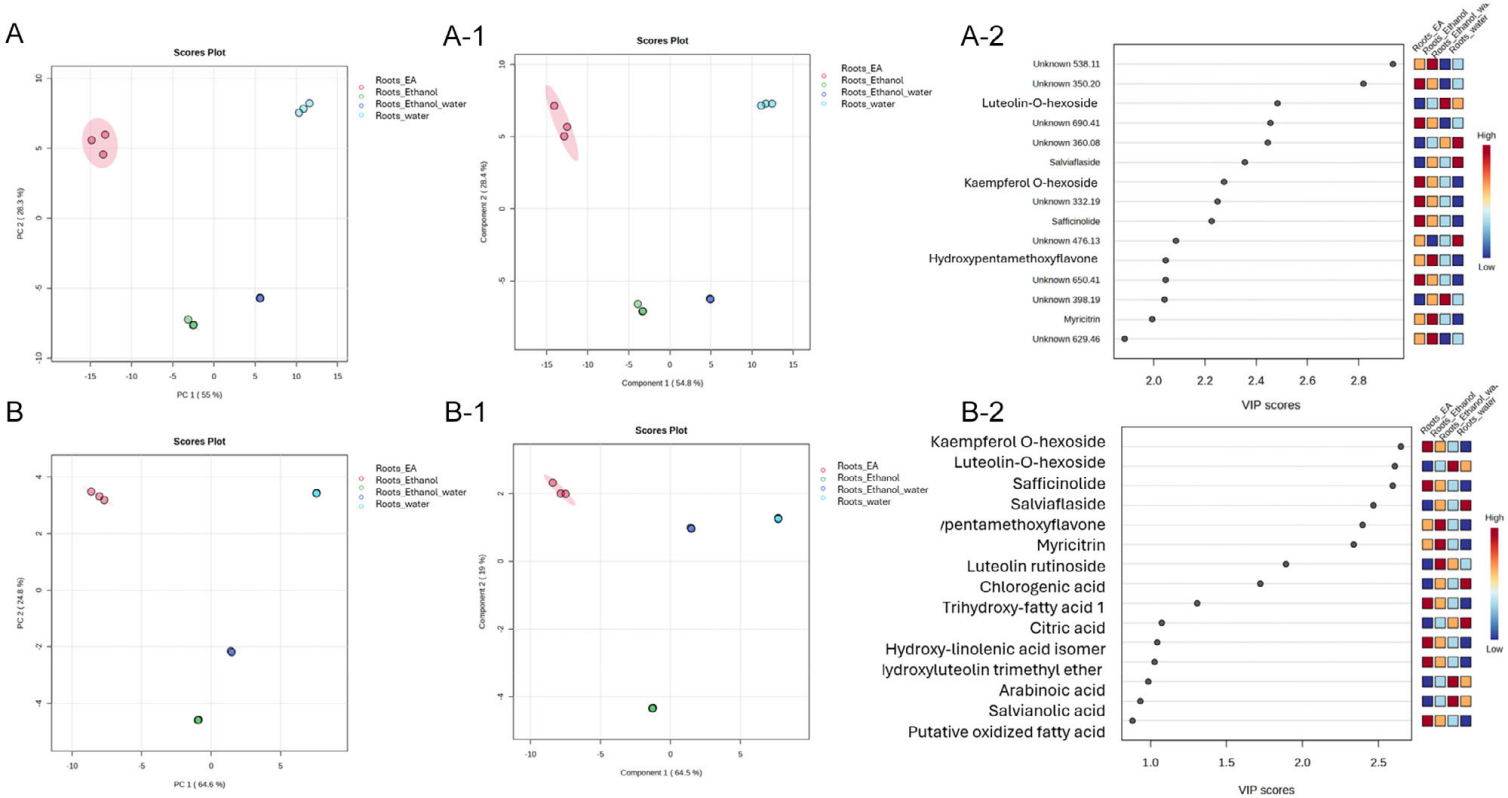
PCA (A) (PERMANOVA: *F*‐value: 1041.1; *R* ^2^: 0.99745; *p*‐value (based on 999 permutations): 0.001), PLS‐DA (A‐1) (*R* ^2^ = 0.9993, *Q* ^2^_cum_ = 0.9952, permutation test *p * =  0.001) analysis and 15 important features (VIP  >  1) (A‐2) obtained from all detected features of *Salvia heldreichiana* extracts from **roots**; PCA (B) (*F*‐value: 2796.7; *R* ^2^: 0.99905; *p*‐value (based on 999 permutations): 0.001), PLS‐DA (B‐1) (*R* ^2^ = 0.9993, *Q* ^2^_cum_ = 0.9982, permutation test *p * =  0.001) analysis and 15 important features (VIP >  1) (B‐2) obtained from identified compounds of *Salvia heldreichiana* extracts from aerial parts (AE) in negative ionization mode.

**FIGURE 10:**
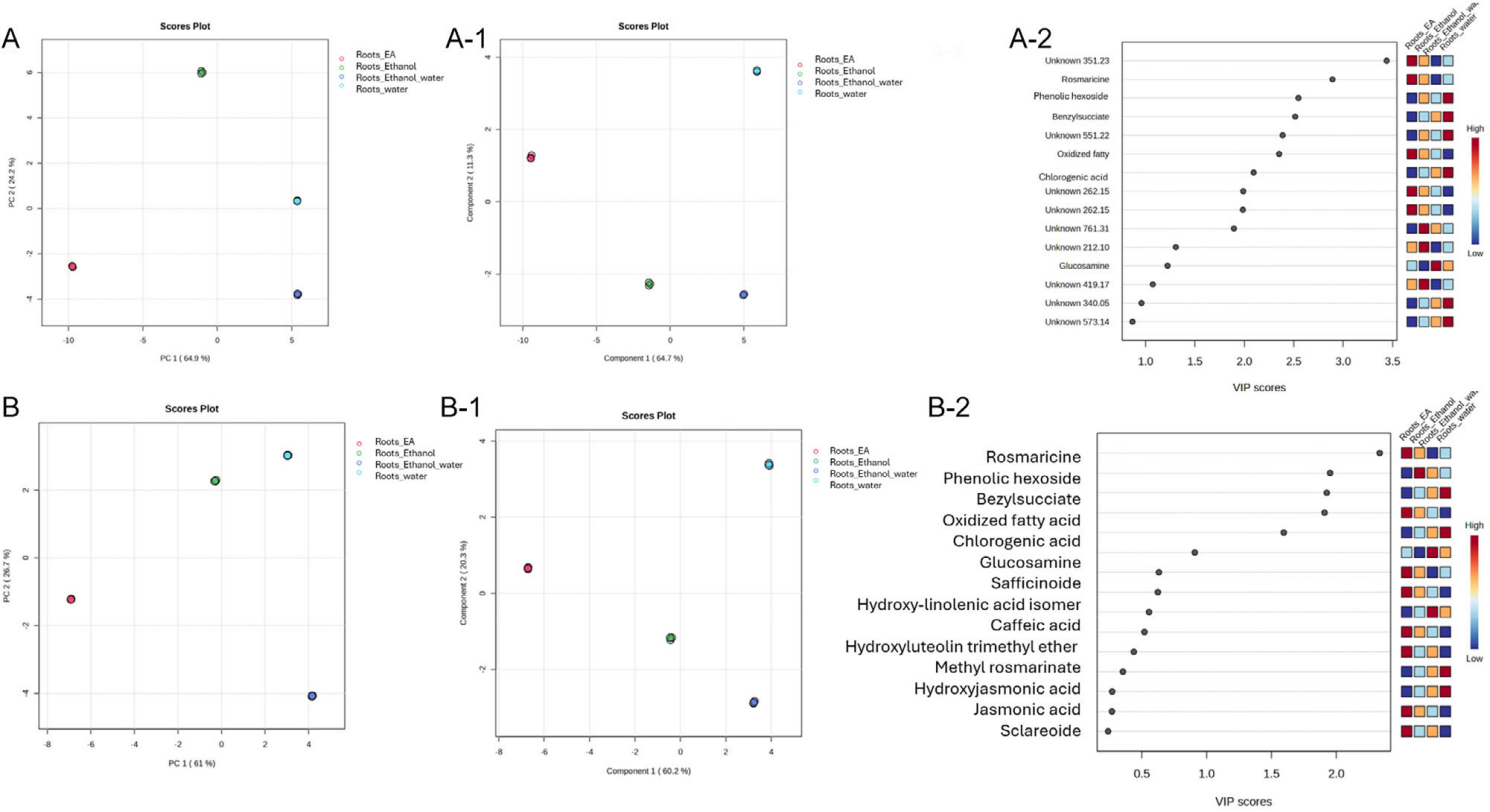
PCA (A) (PERMANOVA: *F*‐value: 78,111; *R* ^2^: 0.99997; *p*‐value (based on 999 permutations): 0.001), PLS‐DA (A‐1) (*R* ^2^ = 0.9999, *Q* ^2^_cum_ = 0.99991, permutation test *p* =  0.001) analysis and 15 important features (VIP   >  1) (A‐2) obtained from all detected features of *Salvia heldreichiana* extracts from roots; PCA (B) (*F*‐value: *F*‐value: 136,380; *R* ^2^: 0.99998; *p*‐value (based on 999 permutations): 0.001), PLS‐DA (B‐1) (*R* ^2^ = 0.9999, *Q* ^2^_cum_ = 0.9998, permutation test *p * =  0.001) analysis and 15 important features (VIP  >  1) (B‐2) obtained from identified compounds of *Salvia heldreichiana* extracts from aerial parts (AE) in positive ionization mode.

For better understanding of which metabolites were responsible for clustering, a supervised PLS‐DA was performed separately for each *Salvia* species, plant organ, and ionization mode. The initial models included all detected molecular features and showed clear discrimination among the four solvent extracts (Figures 3–10A). Variable Importance in Projection (VIP analysis revealed that the discrimination was primarily driven by mainly unknown metabolites, which exhibited the highest VIP scores (>1.0) (Figures 3–10A‐2). Although these features substantially contributed to sample classification, their chemical and biological significance could not be interpreted. Therefore, a second PLS‐DA was performed using only annotated metabolites (Figures 3–10B‐1). Despite excluding the unknown features, the extracts remained clearly separated according to solvent, demonstrating that the observed discrimination was also supported by identified metabolites. In aerial part and roots of *S. tomentosa and S. heldreichiana*, the annotated metabolites with high VIP score belonged to the phenylpropanoid, flavonoid, and terpenoid pathways (Figures 3–10B‐2).

In aerial part of *S. tomentosa*, these compounds included flavonoid glycosides (luteolin, apigenin, chrysoeriol, eupatolin, and hesperetin derivatives), phenolic acids (salvianolic acid), and phenolic diterpenes (carnosic acid and rosmanol) (Figures 3B‐2 and 4B‐2). In roots, carnosic acid, rosmanol, salvianolic acids, and methyl rosmarinate contributed markedly to solvent discrimination (Figures 5B‐2 and 6B‐2). The presence of these compounds among the VIP metabolites indicates that differences in solvent polarity strongly influenced the extraction efficiency of antioxidants.

In *S. heldreichiana*, both aerial and root extracts were differentiated by phenolic acids, flavonoid glycosides, and terpenoid compounds (Figures 7−10B‐2). Important annotated compounds included myricitrin, salvianolic acid derivatives, luteolin‐O‐hexoside, and kaempferol‐O‐hexoside (Figures 7B‐2 and 8B‐2). The root extracts showed a comparatively strong contribution from phenolic acids and organic acids, including chlorogenic acid, caffeic acid, salvianolic acid derivatives, and citric acid, as well as flavonoid derivatives such as kaempferol‐O‐hexoside, luteolin‐O‐hexoside, and hydroxy‐pentamethoxyflavone (Figures 9B‐2 and 10B‐2). These compounds may contribute to the antioxidant, antimicrobial, and enzyme‐inhibitory properties of the extracts.

These results demonstrate that both *S. heldreichiana* and *S. tomentosa* showed strong variation in metabolite profiles across plant parts and extraction solvents.

Tables S1 and S2 summarize the presence of detected metabolites in negative and positive ionization modes in both *Salvia* species. Solvent polarity played a critical role in compound recovery. Ethanol and ethanol/water solvents extracted the highest number of metabolites from both species and tissues. Ethanol has intermediate polarity, coupled with the modifying effect of water in the ethanol/water mixture, and appeared to enhance the solubilization of both polar and semipolar phytochemicals. EA, as a less‐polar solvent, extracted only fewer compounds. Water alone was the least effective solvent, particularly in aerial tissue extracts. The aerial parts of *S. tomentosa* presented a wider metabolite profile compared to *S. heldreichiana* extracted with ethanol and ethanol/water. These solvents were the most efficient, extracting the highest number of compounds, including amino and organic acids, various phenolic, and unknown metabolites.

Root extracts from both *Salvia* species contained a higher number of detected compounds than the aerial parts. Ethanol and ethanol/water extracted a broad range of compounds belonging to different chemical classes. EA extracted a limited number of compounds, especially in *S. heldreichiana*. Water showed relatively better extraction ability in root extracts than aerial parts, potentially reflecting higher water‐soluble compounds in roots.

Several compounds were detected in most extracts. These compounds included luteolin, a flavonoid known for its anti‐inflammatory and antioxidant activities, found in nearly all extracts. Rosmarinic acid is an ester of caffeic acid commonly found in *Salvia* species and known for its potent antioxidant activity. Salvianolic acid is a caffeic acid trimer with cardiovascular and neuroprotective potential, consistently detected across solvent systems. Hydroxybenzoic and dihydroxybenzoic acids, which are phenolic acids, play roles in plant defense and the regulation of oxidative stress.

**FIGURE 11:**
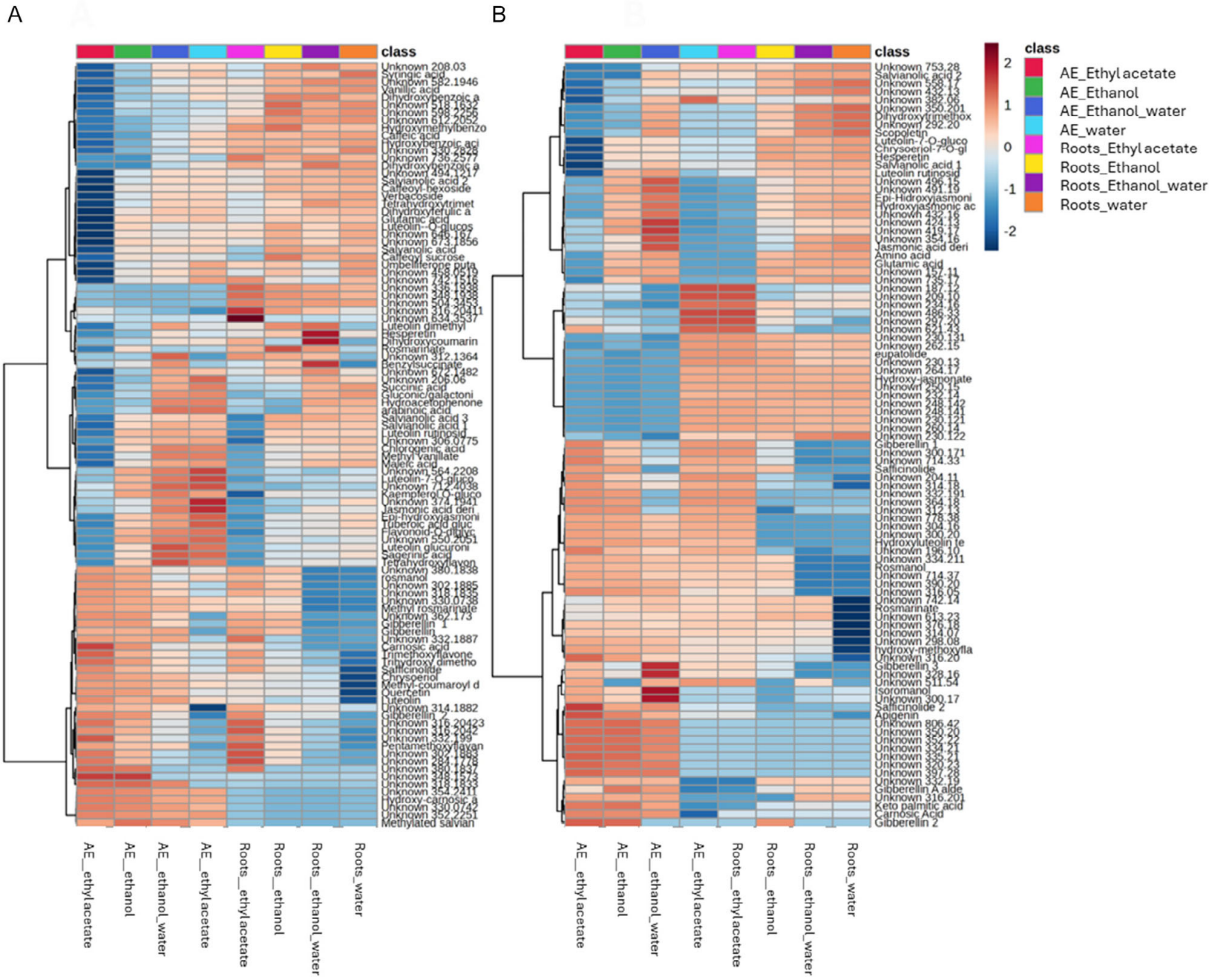
Heat map of aerial parts and root extracts of *Salvia tomentosa* in negative (A) and positive (B) ionization mode. Hierarchical clustering based on Euclidean distance and Ward's linkage method was applied to cluster samples (horizontal axis) and features (vertical axis).

**FIGURE 12:**
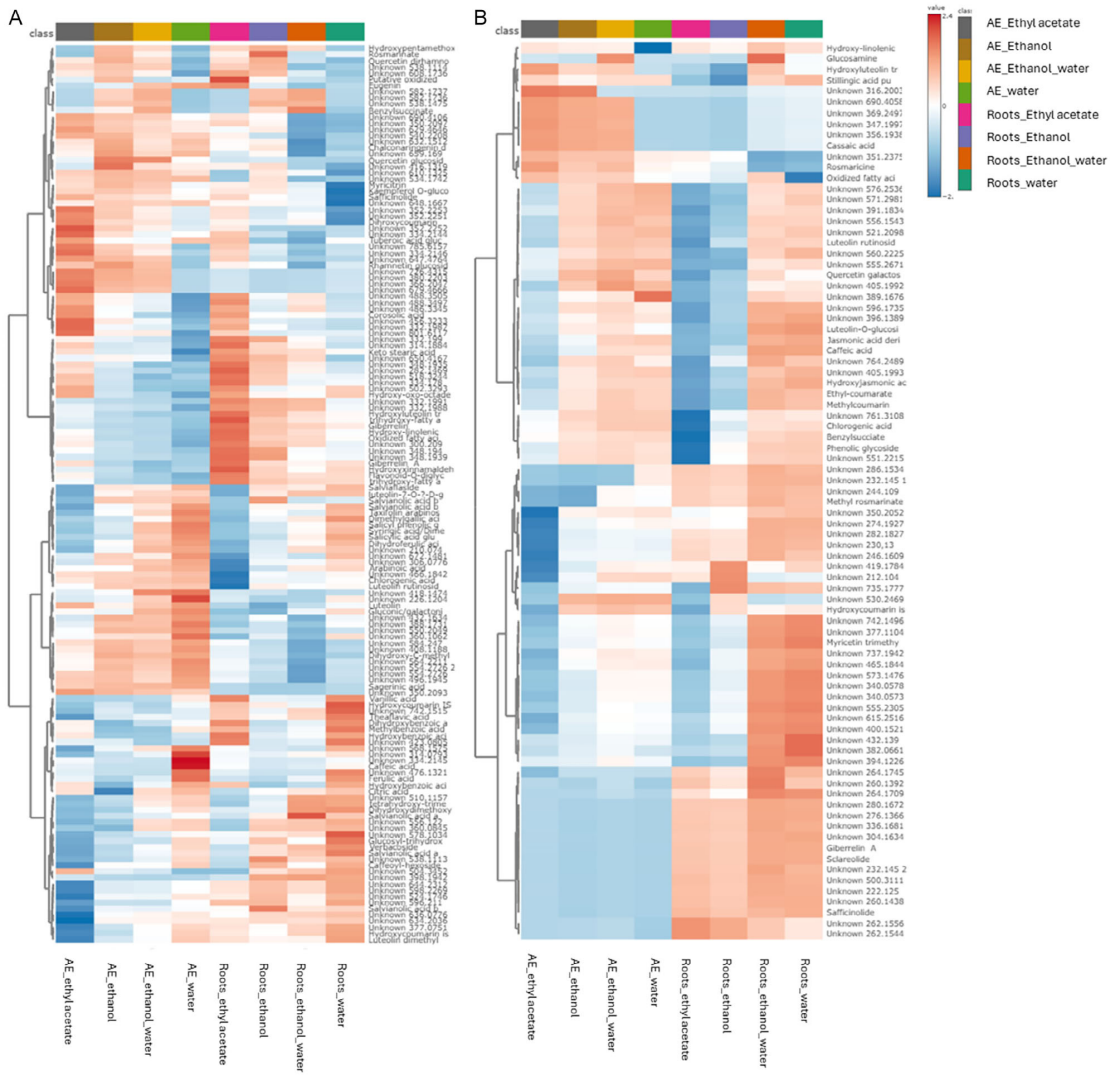
Heat map of aerial parts and root extracts of *Salvia heldreichiana* in negative (A) and positive (B) ionization mode. Hierarchical clustering based on Euclidean distance and Ward's linkage method was applied to cluster samples (horizontal axis) and features (vertical axis).

*S. tomentosa* exhibited greater phytochemical profile than *S. heldreichiana*, particularly in aerial parts. However, many key metabolites were shared between the two species. Hierarchical clustering heatmap analysis (Figure 11) provided a semiquantitative overview of metabolite profiles based on their relative abundances, with compounds ranked according to an ANOVA test (*p* ≤ 0.05), thereby highlighting solvent‐dependent variations in metabolite concentrations for *S. tomentosa* in negative (Figure 11A) and positive (Figure 11B) ionization modes and *S. heldreichiana* in negative (Figure 12A) and positive (Figure 12B) ionization modes**.**

### 3.3 Antioxidant Effects

Plant natural metabolites exhibit antioxidant activity through multiple mechanisms, including free‐radical scavenging, metal‐ion chelation (e.g., iron and copper), and inhibition of enzymes involved in radical generation [54, 55]. *Salvia* has attracted considerable attention due to their richness in bioactive constituents and particularly phenolic and flavonoid compounds, which are closely associated with strong antioxidant potential [52, 56, 57]. *S*. *heldreichiana* has been previously studied for its antioxidant activity as well as for its essential oils and phenolic content [28, 29, 48]. The current study expands on this by evaluating the aerial parts and the roots EA, ethanol, ethanol/water, and water extracts across multiple antioxidant assays. Both *S*. *heldreichiana* and *S*. *tomentosa* exhibited the highest antioxidant capacity in almost all tested assays (DPPH, ABTS, CUPRAC, FRAP, MCA, and PBD). The results are given in Table 2, which illustrates that the antioxidant activity varied considerably among the species, plant parts, and solvent systems. The *S*. *heldreichiana* aerial parts water extract possessed the highest antioxidant capacity in CUPRAC (751.00 mg TE/g) > FRAP (610.05 mg TE/g) > ABTS (522.13 mg TE/g) > DPPH (332.83 mg TE/g) > MCA 21.36 > PBD 3.94 mmol TE/g compared to the other extracts. The root extracts of *S*. *heldreichiana* followed a similar trend, with the highest antioxidant potential observed in the ethanol/water extract. However, the EA extracts of both parts of the plant showed the lowest antioxidant activity in all the methods taken together. The current finding is unlike previous reports [48], which indicated that EA extracts of *S*. *heldreichiana* possessed a higher antioxidant activity, especially in the FRAP assay.

**TABLE 2:** Antioxidant properties of the tested extracts.

*Salvia* species	Parts	Extracts	DPPH, mg TE/g	ABTS, mg TE/g	CUPRAC, mg TE/g	FRAP, mg TE/g	MCA, mg EDTAE/g	PBD, mmol TE/g
*S. heldreichiana*	Aerial parts	Ethyl acetate	59.51 ± 0.95^j^	143.90 ± 0.61^i^	134.88 ± 4.52^i^	85.69 ± 8.81^kl^	7.71 ± 1.94^fg^	2.17 ± 0.17^h^
		Ethanol	162.08 ± 5.62^i^	323.52 ± 6.83^efg^	444.54 ± 7.66^f^	380.89 ± 8.30^h^	3.29 ± 0.88^i^	2.82 ± 0.05^fg^
		Ethanol/water	305.75 ± 6.08^b^	427.30 ± 4.01c	656.75 ± 8.55^b^	547.31 ± 4.78^c^	14.38 ± 0.15^bc^	3.87 ± 0.32^ab^
		Water	332.83 ± 6.05^a^	522.13 ± 6.85^a^	738.04 ± 7.17^a^	582.87 ± 6.44^b^	21.36 ± 0.09^a^	3.94 ± 0.03^a^
	Roots	Ethyl acetate	47.36 ± 0.80^j^	93.34 ± 1.76^j^	150.78 ± 3.67^i^	97.91 ± 4.26^k^	15.98 ± 0.57^b^	2.27 ± 0.08^h^
		Ethanol	208.02 ± 6.34^ef^	342.86 ± 12.00^e^	518.88 ± 12.67^e^	449.91 ± 8.02^g^	5.81 ± 1.04^gh^	3.34 ± 0.13^cde^
		Ethanol/water	311.04 ± 2.22^b^	513.63 ± 5.89^a^	751.00 ± 10.19^a^	610.05 ± 21.93^a^	15.07 ± 0.53^b^	3.72 ± 0.07^abc^
		Water	237.46 ± 10.27^d^	393.90 ± 5.71^d^	623.63 ± 7.58^c^	523.39 ± 7.28^d^	12.30 ± 0.26^cd^	3.49 ± 0.02^bcd^
*S. tomentosa*	Aerial parts	Ethyl acetate	22.84 ± 0.59^k^	53.30 ± 1.04^k^	105.70 ± 0.85^j^	68.54 ± 0.78^l^	21.17 ± 0.62^a^	2.44 ± 0.13^gh^
		Ethanol	165.02 ± 10.93^hi^	246.51 ± 14.89^h^	463.79 ± 11.10^f^	386.96 ± 3.90^h^	10.75 ± 0.48^de^	3.81 ± 0.27^ab^
		Ethanol/water	283.88 ± 4.07^c^	470.22 ± 13.16^b^	593.67 ± 12.07^d^	485.56 ± 5.52^e^	8.61 ± 0.41^ef^	3.82 ± 0.17^ab^
		Water	214.48 ± 6.97^e^	311.56 ± 13.22^fg^	578.58 ± 9.51^d^	474.62 ± 3.23^ef^	14.93 ± 0.41^b^	3.48 ± 0.02^bcd^
	Roots	Ethyl acetate	18.79 ± 2.22^k^	79.62 ± 4.09^j^	187.50 ± 3.26^h^	150.85 ± 2.24^j^	7.04 ± 1.42^fgh^	2.06 ± 0.17^h^
		Ethanol	63.59 ± 0.07^j^	148.45 ± 0.13^i^	303.37 ± 3.20^g^	222.50 ± 0.74^i^	5.69 ± 0.62^hi^	3.55 ± 0.04^abc^
		Ethanol/water	182.46 ± 4.14^gh^	299.89 ± 13.15^g^	447.21 ± 2.83^f^	397.27 ± 1.49^h^	5.87 ± 0.30^gh^	2.91 ± 0.08^ef^
		Water	193.75 ± 10.63^fg^	331.76 ± 0.80^ef^	521.42 ± 2.22^e^	461.66 ± 4.47^fg^	10.82 ± 0.28^de^	3.10 ± 0.07^def^

*Note*: Values are reported as mean ± SD of three parallel measurements. Different letters indicate significant differences between the tested extracts (*p* < 0.05).

Abbreviations: EDTAE, EDTA equivalent; MCA, metal chelating activity; PBD, phosphomolybdenum; TE, Trolox equivalent.

In the case of *S*. *tomentosa*, aerial parts of ethanol/water were comparatively found to possess the highest antioxidant activity in the following order: CUPRAC (593.67 mg TE/g)  >  FRAP (485.56 mg  TE/g)  >  ABTS (470.22 mg  TE/g)  >  DPPH (283.88 mg  TE/g)  >  MCA (15.07 mg  TE/g)  >  PBD 3.82 mmol  TE/g. The aerial part water extract followed a similar trend. The highest antioxidant activity may be attributed to their higher phenolic content, as reported in previous studies [58, 59, 60]; phenolic compounds are largely responsible for the antioxidant effect. Similarly, the aerial parts of the EA extract exhibited the highest MCA, measuring 21.17 mg EDTAE/g, consistent with the previous study [61] reporting strong MCA of *S*. *cilicica* EA extract. Similar to *S*. *heldreichiana*, *S*. *tomentosa* also exhibited low antioxidant activity in the EA extracts, particularly in the DPPH and PBD assays, with measured values of 22.84 and 2.44 mmol TE/g, respectively.

**FIGURE 13:**
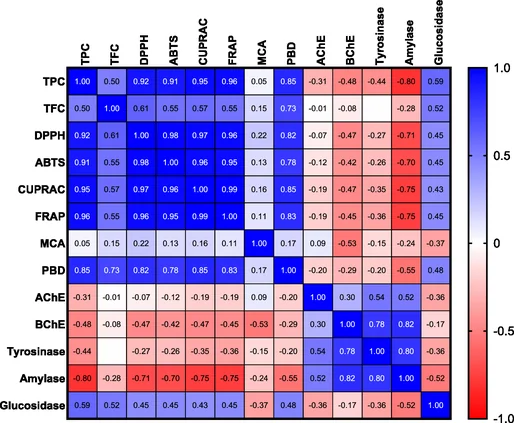
Pearson correlation between the total phenolic/flavonoid contents and biological activities.

Taken together, the antioxidant activity of the tested *Salvia* species increased in line with the polarity of the extraction solvents used. This can be attributed to the enhanced extraction of phenolic compounds and flavonoids from the plant material. To verify this, we carried out a Pearson correlation analysis between total phenolic/flavonoid contents and antioxidant activities. As shown in Figure 13, the antioxidant activities exhibited a strong correlation with the total phenolic and flavonoid contents in the extracts, with the exception of metal‐chelating activity. Based on this observation, the metal‐chelating capacity is likely due to nonphenolic chelators, such as peptides and polysaccharides. In the literature, numerous studies have similarly documented a strong association between the total phenolic/flavonoid content and the antioxidant activity of species belonging to the *Salvia* genus [62, 63, 64]. Therefore, we suggested using polar solvents to produce extracts from species of the *Salvia* genus for the development of functional applications.

### 3.4 Enzyme Inhibition Activity

In the present study, the enzyme‐inhibitory activity of extracts from *S*. *heldreichiana* and *S*. *tomentosa* was evaluated, and the results are presented in Table 3, comparing their inhibitory effects with those of standard drugs at the same concentration. AChE and BChE inhibitory activities were found to differ considerably among species, plant parts, and extraction solvents. In *S*. *heldreichiana*, root extracts demonstrated relatively higher AChE inhibitory activity, particularly in ethanol extracts (2.88 mg GALAE/g) and ethanol/water (2.75 mg GALAE/g). However, the ethanol extract of the aerial part showed no activity. BChE inhibitory activity was found to be highest in the ethanol extract of the aerial part, measuring 3.65 mg GALAE/g, followed by the root extracts, measuring 3.49 mg GALAE/g. In contrast, the water extracts of both parts of the plant showed the least AChE and BChE inhibition activity. Our significantly higher inhibitory activity compared to prior findings [31] demonstrates that the extraction solvent is a decisive factor for isolating bioactive principles from *S. heldreichiana*. In *S*. *tomentosa*, both parts’ extracts showed variable activity, with the highest activity observed in the aerial parts’ ethanol extracts for AChE inhibition, measuring 2.93 mg GALAE/g, and the second highest in BChE inhibition, measuring 3.22 mg GALAE/g. Similar to *S*. *heldreichiana* and *S*. *tomentosa*, both tested parts of the water extracts exhibited the lowest AChE and BChE inhibition activity. The *Salvia* genus has been reported to have physiological effects on the nervous system. Several studies have indicated that some of its species (*Salvia chrysophylla*, *Salvia microstegia*, and *Salvia lavandulifolia*, among others) show marked inhibitory activity against the enzymes acetylcholinesterase (AChE) and butyrylcholinesterase (BChE) [52, 56, 65].

**TABLE 3:** Enzyme inhibitory effects of the tested extracts.

*Salvia* species	Parts	Extracts	AChE, mg GALAE/g	BChE, mg GALAE/g	Tyrosinase, mg KAE/g	Amylase, mmol ACAE/g	Glucosidase, mmol ACAE/g
*S. heldreichiana*	Aerial parts	Ethyl acetate	2.72 ± 0.17^ab^	2.28 ± 0.15^cd^	47.84 ± 4.09^cd^	0.44 ± 0.01^d^	2.68 ± 0.05^bcd^
		Ethanol	na	3.65 ± 0.03^a^	54.90 ± 3.69^ab^	0.39 ± 0.01^e^	2.67 ± 0.01^cd^
		Ethanol/water	2.64 ± 0.01^b^	1.81 ± 0.11^e^	52.57 ± 0.60^abc^	0.30 ± 0.01^g^	2.61 ± 0.04^d^
		Water	1.13 ± 0.01^c^	0.83 ± 0.08^fg^	43.17 ± 1.33^d^	0.05 ± 0.01^h^	2.71 ± 0.02^abc^
	Roots	Ethyl acetate	2.73 ± 0.13^ab^	2.61 ± 0.20^cd^	56.40 ± 0.32^ab^	0.58 ± 0.02^b^	na
		Ethanol	2.88 ± 0.05^a^	3.49 ± 0.11^a^	51.20 ± 0.58^bc^	0.46 ± 0.01^cd^	2.44 ± 0.01^e^
		Ethanol/water	2.75 ± 0.03^ab^	2.07 ± 0.01^de^	56.84 ± 0.37^ab^	0.30 ± 0.01^g^	na
		Water	1.11 ± 0.08^c^	1.01 ± 0.11^f^	22.55 ± 2.40^e^	0.05 ± 0.01^h^	2.69 ± 0.03^abc^
*S. tomentosa*	Aerial parts	Ethyl acetate	2.62 ± 0.19^b^	2.62 ± 0.61^c^	58.17 ± 0.49^a^	0.62 ± 0.01^a^	na
		Ethanol	2.93 ± 0.01^a^	3.22 ± 0.07^ab^	53.70 ± 0.35^abc^	0.45 ± 0.01^cd^	2.47 ± 0.05^e^
		Ethanol/water	2.88 ± 0.02^a^	1.97 ± 0.08^e^	52.98 ± 4.71^abc^	0.37 ± 0.01^e^	2.77 ± 0.03^a^
		Water	0.51 ± 0.05^d^	na	28.26 ± 1.38^e^	0.06 ± 0.01^h^	2.40 ± 0.02^ef^
	Roots	Ethyl acetate	2.89 ± 0.06^a^	2.80 ± 0.12^bc^	53.23 ± 1.74^abc^	0.57 ± 0.01^b^	na
		Ethanol	na	3.42 ± 0.09^a^	53.59 ± 0.50^abc^	0.47 ± 0.01^c^	2.34 ± 0.01^f^
		Ethanol/water	2.92 ± 0.02^a^	2.69 ± 0.03^bc^	58.55 ± 0.12^a^	0.34 ± 0.01^f^	2.76 ± 0.02^ab^
		Water	0.57 ± 0.02^d^	0.45 ± 0.06^gh^	24.45 ± 2.65^e^	0.06 ± 0.01^h^	2.32 ± 0.01^f^

*Note*: Values are reported as mean ± SD of three parallel measurements. Different letters indicate significant differences between the tested extracts (*p* < 0.05).

Abbreviations: ACAE, acarbose equivalent; GALAE, galantamine equivalent; KAE, kojic acid equivalent; na, not active.

In the case of tyrosinase inhibition activity, both *S. heldreichiana and S. tomentosa*, when tested, exhibited the highest anti‐tyrosinase activity in all extracts, except for the water extracts, which consistently showed comparatively lower activity across the evaluated assay. In *S. heldreichiana* roots, ethanol/water and EA extracts exhibited comparable activity, with values of 56.84 and 56.40 mg KAE/g, respectively. The higher anti‐tyrosinase activity compared to the previous result of Yilmaz et al. [31] conclusively demonstrates that the choice of extraction solvent is the decisive factor in recovering these potent bioactive compounds from *S. heldreichiana*. This study provides the first evaluation of the tyrosinase inhibitory activity from *S*. *tomentosa* extract, contrasting with previous research that has focused solely on the plant's essential oils [66]. In *S*. *tomentosa*, extracts obtained from different EA of aerial parts and ethanol/water of roots showed the highest and comparable activity, with values of 58.17 and 58.55 mg KAE/g, respectively. The potent tyrosinase inhibition observed in this study is strongly supported by previous biological research demonstrating that *S. tomentosa* extracts can reduce key inflammatory and melanogenic markers, such as PGE2 and L‐dopa turnover, in skin cells [67].

α‐Amylase and α‐glucosidase are key digestive enzymes involved in the breakdown of dietary carbohydrates into simpler sugars and glucose. Inhibition of these enzymes slows carbohydrate digestion and delays glucose absorption in the intestine [68]. Among the evaluated extracts, EA extracts from both plant parts in both species, *S*. *heldreichiana* and *S*. *tomentosa,* exhibited the highest α‐amylase inhibition activity, demonstrating superior activity relative to the corresponding extracts obtained from the same parts of each plant. *S*. *heldreichiana* EA extracts of the roots exhibited the highest α‐amylase inhibition activity, measuring 0.58 mmol ACAE/g, followed by EA aerial part extracts, measuring 0.44 mmol ACAE/g. Similarly, in *S*. *tomentosa*, the EA extract from aerial parts and then roots showed the highest anti‐α‐amylase activity, measuring inhibition value 0.62 and 0.75 mmol ACAE/g, respectively. This indicates consistently superior activity of EA extracts across the two species. Mahdi et al. [69] similarly reported that the ethyl acetate fraction of *S. officinalis* hydro‐methanolic decoction exhibited the highest antidiabetic activity through strong α‐amylase and α‐glucosidase inhibition, which was attributed to its high phenolic content.

In the case of the α‐glucosidase inhibition activity, *S*. *heldreichiana* water extracts of aerial parts (2.71 mmol ACAE/g) and roots (2.69 mmol ACAE/g) generally exhibited the highest inhibition activity among the evaluated samples. In contrast, for *S*. *tomentosa*, the Ethanol/Water extracts of both parts showed the highest α‐glucosidase inhibition activity among the tested extracts, measuring 2.77 and 2.76 mmol ACAE/g, respectively. On the other hand, their EA extracts showed no α‐glucosidase inhibition activity. To the best of our knowledge, the current study is the first to report the α‐amylase and α‐glucosidase inhibition activities of *S*. *heldreichiana* and *S*. *tomentosa*, thus highlighting a new, unexplored antidiabetic potential of these species that conforms to the known bioactivity of the *Salvia* genus.

Across all five enzyme targets, the inhibitory pattern tracked closely with solvent polarity and the class of metabolites each solvent could recover. Ethanolic extracts, especially from roots, gave the strongest AChE and BChE inhibition, consistent with ethanol's ability to solubilize some compounds, including rosmarinic acid, that can dock into the aromatic‐lined catalytic and peripheral sites of cholinesterases [70]. The best tyrosinase and amylase inhibition was provided by more lipophilic ethyl acetate extracts, reflecting the affinity of this solvent for flavonoid aglycones and moderately nonpolar phenolics capable of chelating tyrosinase's dicopper center [71] or engaging hydrophobic pockets near amylase's substrate‐binding cleft [62]. Glucosidase inhibition moderately correlated with the total phenolic and flavonoid contents, as reported in Figure 13. Overall, this indicates that each enzyme's inhibition profile is shaped by a genuine chemical match between the metabolites a solvent extracts and the structural demands of that enzyme's active site, a relationship future work could confirm through targeted metabolomic profiling of the most active fractions.

Carbonic anhydrase isozymes play a central role in fundamental physiological processes, including acid–base balance, respiration, electrolyte secretion, and metabolic regulation [72, 73, 74]. Dysregulation of specific carbonic anhydrase isoforms has been associated with several pathological conditions such as glaucoma, epilepsy, obesity, cancer, and neurodegenerative disorders. For this reason, carbonic anhydrase inhibitors are widely used in clinical practice for the treatment of glaucoma, edema, altitude sickness, and certain neurological diseases [72, 75]. However, most clinically approved inhibitors, including sulfonamide‐based drugs such as acetazolamide, are often associated with adverse side effects and limited isoform selectivity [72, 76]. The inhibition activities of *S. tomentosa* and *S. heldreichiana* extracts against human carbonic anhydrase isoenzymes I and II were evaluated based on their IC_50_ values, as summarized in Table 4. All tested extracts exhibited concentration‐dependent inhibition effects with high determination coefficients (*r* ^2^ = 0.93–0.99), indicating reliable dose–response relationships. Regarding hCA I inhibition, the ethanol/water extract of *S. tomentosa* aerial parts showed the strongest activity with 1.39 ±  0.14  µg/mL IC_50_ value, followed by its ethyl acetate and aqueous extracts. Several extracts, particularly from the aerial parts of both species, demonstrated stronger inhibitory effects than the standard drug acetazolamide (IC_50_ = 4.60  ±  0.72  µg/mL). In general, aerial part extracts exhibited more pronounced hCA I inhibitory activity compared to root extracts. In terms of hCA II inhibition, remarkable activity was observed for the ethanol/water extract of *S. heldreichiana* aerial parts, which displayed the lowest with 0.93  ±  0.03 µg/mL IC_50_ value. Additionally, the ethanol extract of *S. heldreichiana* roots and the aqueous extract of *S. tomentosa* roots showed notable inhibitory effects. Most extracts exhibited stronger hCA II inhibition than acetazolamide (IC_50_ = 10.00  ±  1.09  µg/mL). Overall, ethanol/water extracts demonstrated superior inhibitory activities against both isoenzymes, suggesting that polar bioactive constituents may contribute to the observed carbonic anhydrase inhibitory activity. Aerial part extracts generally exhibited stronger inhibition against hCA I, whereas root extracts tended to show comparatively enhanced inhibition against hCA II, particularly in *S. tomentosa*. The observed variations among plant parts and extraction solvents suggest distinct isoenzyme‐specific inhibition profiles for both species.

**TABLE 4:** Carbonic anhydrase inhibitory effects of the tested extracts (IC_50_, µg/mL).

Extract name	Parts	Extracts	hCA I		hCA II	
			IC_50_	*r* ^2^	IC_50_	*r* ^2^
*S. toentosa*	Aerial parts	EA	2.87 ± 0.46	0.9665	7.37 ± 0.87	0.9578
		Ethanol	11.60 ± 1.07	0.9780	16.84 ± 1.23	0.9900
		Ethanol/water	1.39 ± 0.14	0.9729	11.99 ± 1.08	0.9554
		Water	3.11 ± 0.49	0.9836	10.25 ± 1.0	0.9564
	Roots	EA	5.68 ± 0.75	0.9430	18.48 ± 1.27	0.9751
		Ethanol	8.13 ± 0.93	0.9919	3.44 ± 0.54	0.9847
		Ethanol/Water	4.63 ± 0.67	0.9969	3.23 ± 0.51	0.9288
		Water	4.23 ± 0.63	0.9855	2.86 ± 0.46	0.9708
*S. heldreichiana*	Aerial parts	EA	3.85 ± 0.58	0.9884	2.52 ± 0.40	0.9765
		Ethanol	3.64 ± 0.56	0.9792	2.81 ± 0.45	0.9651
		Ethanol/water	3.50 ± 0.54	0.9693	0.93 ± 0.03	0.9884
		Water	6.51 ± 0.81	0.9955	3.21 ± 0.51	0.9815
	Roots	EA	3.59 ± 0.56	0.9751	11.80 ± 1.07	0.9989
		Ethanol	4.03 ± 0.61	0.9882	1.41 ± 0.15	0.9499
		Ethanol/water	3.71 ± 0.57	0.9747	3.43 ± 0.54	0.9861
		Water	4.06 ± 0.61	0.9907	2.90 ± 0.46	0.9734
Standard drug (acetazolamide)			4.60 ± 0.72	0.9916	10.00 ± 1.09	0.9921

The strong inhibitory activities observed for the ethanol/water extracts may be associated with their relatively high contents of caffeic acid, rosmarinic acid, and salvianolic acid derivatives, as identified by the LC–MS/MS analysis. These compounds contain catechol and carboxylate functional groups, which have been reported to facilitate interactions with the zinc‐bound water system and to form hydrogen bonds within the carbonic anhydrase active site [77, 78, 79]. In addition, the presence of luteolin‐ and chrysoeriol‐based flavonoid glycosides may contribute to the stabilization of enzyme–inhibitor complexes through aromatic and peripheral binding interactions [80]. Moreover, the detection of scopoletin and related coumarin derivatives may indicate the possible involvement of channel‐blocking mechanisms in enzyme inhibition [81]. Overall, the combined presence of these bioactive compounds in the most active extracts, particularly in the aerial parts of *S. heldreichiana* and the ethanol/water fractions of *S. tomentosa*, may partially explain the low IC_50_ values and the observed isoenzyme‐selective inhibition profiles. However, further studies using isolated compounds are required to confirm their individual contributions to the observed inhibitory activity.

The carbonic anhydrase inhibitory activities observed for *S. tomentosa* and *S. heldreichiana* extracts are consistent with, and in several cases superior to, those previously reported for *Salvia* species and related Lamiaceae plants [82, 83, 84, 85, 86, 87]. For instance, the ethanol and water extracts of *S. fruticosa* were reported to inhibit hCA II with 14.51  ±  4.07 and 26.87  ±  3.56 µg/mL IC_50_ values, respectively [82]. In contrast, the ethanol/water extracts of *S. heldreichiana* aerial parts in the present study exhibited markedly stronger activity, with an IC_50_ value of 0.93  ±  0.03 µg/mL against hCA II, corresponding to approximately a 10–25‐fold lower IC_50_ value than those reported for previously investigated *Salvia* extracts. Moreover, the ethanol/water extract of *S. tomentosa* aerial parts showed an IC_50_ value of 1.39 ± 0.14 µg/mL against hCA I, which is also substantially lower than those reported for most crude plant extracts [82, 83, 84, 85, 86, 87]. At the compound level, rosmarinic acid has been identified as a potent CA inhibitor, with strong activity against hCA isoenzymes [82], suggesting that it may contribute to the observed inhibitory activity. Therefore, when compared with previously studied *Salvia* species and isolated phenolic compounds, the present extracts, particularly the polar aerial fractions, demonstrate exceptionally high inhibitory potential, which may be associated with their relatively high contents of caffeic acid, rosmarinic acid, and salvianolic acid derivatives, as identified by LC–MS/MS analysis [82, 83, 84, 85, 86, 87].

Although several Salvia species have been investigated for their phytochemical composition and various biological activities, studies focusing on carbonic anhydrase inhibition remain limited. To the best of our knowledge, the inhibitory effects of *S. tomentosa* and *S. heldreichiana* against hCA I and hCA II have not been previously reported. Most available studies have focused on other Salvia species, such as *S. fruticosa* and *S. macrochlamys*, which showed moderate inhibition at 8–30 µg/mL [82, 84]. In contrast, the present study demonstrates that the ethanol/water extracts of *S. tomentosa* and *S. heldreichiana* exhibit remarkably stronger inhibition, with IC_50_ values of 1.39 ± 0.14 µg/mL for hCA I and 0.93 ± 0.03 µg/mL for hCA II. These findings represent the first systematic evaluation of carbonic anhydrase inhibitory activity for these two *Salvia* species and suggest that they may serve as promising sources of bioactive compounds for further investigation as carbonic anhydrase inhibitors.

### 3.5 Antimicrobial Effects

The antimicrobial evaluation demonstrated that *S. tomentosa* exhibits a selective inhibitory profile, with pronounced activity against Gram‐positive bacteria (Table 5) and dermatophytic fungi (Table 6), while showing comparatively weak effects against yeast strains. When compared with *S. heldreichiana*, *S. tomentosa* consistently displayed lower MIC values across most tested microorganisms. Among bacterial strains, *Staphylococcus aureus* was the most susceptible to *S. tomentosa*, with MIC values ranging from 12.5 to 25 µg/mL. In contrast, *S. heldreichiana* inhibited *S. aureus* at higher concentrations (25–50 µg/mL). A similar trend was observed for *Escherichia coli*, for which *S. tomentosa* showed MIC values of 25–50 µg/mL, whereas *S. heldreichiana* required 50–100 µg/mL. Gram‐negative bacteria, including *Pseudomonas aeruginosa* and *Salmonella typhi*, exhibited reduced sensitivity to both extracts. However, *S. tomentosa* generally showed marginally lower MIC values than *S. heldreichiana*.

**TABLE 5:** MIC of *Salvia tomentosa* extracts against yeast, bacterial, and dermatophytes strains.

	Strain (ID)	Minimum inhibitory concentration (MIC)^a^								
		AP‐EA	AP‐EtOH	AP‐EtOH/water	AP‐water	R‐EA	R‐EtOH	R‐EtOH/water	R‐water	Fluconazole, µg/mL
Yeasts	*C. tropicalis* (DBVPG 6184)	>200	125.99 (100–200)	125.99 (100–200)	>200	125.99 (100–200)	125.99 (100–200)	>200	>200	2
	*C. albicans* (DBVPG 6379)	>200	>200	125.99 (100–200)	>200	125.99 (100–200)	125.99 (100–200)	>200	>200	1
	*C. parapsilosis* (DBVPG 6551)	>200	>200	>200	125.99 (100–200)	>200	>200	>200	125.99 (100–200)	4
	*Gram−*									Ciprofloxacin, µg/mL
Bacteria	*E. coli* (ATCC 10536)	31.49 (25–50)	62.99 (50–100)	79.37 (50–100)	>200	31.49 (25–50)	39.68 (25–50)	125.99 (100–200)	>200	<0.12
	*P. aeruginosa* (ATCC15442)	79.37 (50–100)	>200	125.99 (100–200)	39.68 (25–50)	158.74 (100–200)	125.99 (100–200)	79.37 (50–100)	125.99 (100–200)	1.23 (0.98–1.95)
	*S. typhi* (ATCC 15442)	125.99 (100–200)	>200	79.37 (50–100)	39.68 (25–50)	125.99 (100–200)	>200	62.99 (50–100)	39.68 (25–50)	0.38 (0.24–0.49)
	*Gram+*									
	*B. cereus* (ATCC 12826)	62.99 (50–100)	>200	>200	>200	>200	>200	>200	>200	<0.12
	*B. subtilis* (PERU 6)	62.99 (50–100)	39.68 (25–50)	62.99 (50–100)	79.37 (50–100)	62.99 (50–100)	39.68 (25–50)	62.99 (50–100)	79.37 (50–100)	<0.12
	*S. aureus.* (ATCC 6538)	31.49 (25–50)	15.74 (12.5–25)	31.49 (25–50)	62.99 (50–100)	39.68 (25–50)	31.49 (25–50)	39.68 (25–50)	>200	0.62 (0.98–0.49)
										Griseofulvin, µg/mL
Dermatophytes	*T. mentagrophytes* (CCF 4823)	62.99 (50–100)	62.99 (50–100)	79.37 (50–100)	125.99 (100–200)	62.99 (50–100)	>200	>200	158.74 (100–200)	2.52 (2–4)
	*T. tonsurans* (CCF 4834)	79.37 (50–100)	125.99 (100–200)	>200	>200	79.37 (50–100)	158.74 (100–200)	>200	>200	0.198 (0.125–0.25)
	*T. rubrum* (CCF 4933)	39.68 (25–50)	31.49 (25–50)	>200	>200	39.68 (25–50)	125.99 (100–200)	>200	>200	3.175 (2–4)
	*A. quadrifidum* (CCF 5792)	62.99 (50–100)	39.68 (25–50)	125.99 (100–200)	79.37 (50–100)	79.37 (50–100)	>200	79.37 (50–100)	>200	>8
	*T. erinacei* (CCF 5930	39.68 (25–50)	19.84 (12.5–25)	125.99 (100–200)	>200	31.49 (25–50)	79.37 (50–100)	79.37 (50–100)	>200	3.174 (2–4)
	*A. gypseum* (CCF 6261)	31.49 (25–50)	31.49 (25–50)	158.74 (100–200)	125.99 (100–200)	39.68 (25–50)	>200	125.99 (100–200)	125.99 (100–200)	1.587 (1–2)
	*A. currey* (CCF 5207)	31.49 (25–50)	62.99 (50–100)	62.99 (50–100)	158.74 (100–200)	62.99 (50–100)	125.99 (100–200)	>200	>200	>8
	*A. insingulare* (CCF 5417)	39.68 (25–50)	39.68 (25–50)	31.49 (25–50)	158.74 (100–200)	125.99 (100–200)	>200	125.99 (100–200)	158.74 (100–200)	>8

a MIC values are reported as geometric means of three independent replicates (*n* = 3). MIC range concentrations are reported within brackets.

**TABLE 6:** MIC of *Salvia heldreichiana* extracts against yeast, bacterial, and dermatophytes strains.

	Strain (ID)	Minimum inhibitory concentration (MIC)^a^								
		AP‐EA	AP‐EtOH	AP‐EtOH/water	AP‐water	R‐EA	R‐EtOH	R‐EtOH/water	R‐water	Fluconazole, µg/mL
	*C. tropicalis* (DBVPG 6184)	158.74 (100–200)	>200	>200	>200	>200	158.74(100–200)	>200	>200	2
Yeasts	*C. albicans* (DBVPG 6379)	>200	>200	>200	>200	>200	>200	>200	>200	1
	*C. parapsilosis* (DBVPG 6551)	>200	>200	>200	158.74 (100–200)	>200	158.74 (100–200)	>200	>200	4
	*Gram−*									Ciprofloxacin, µg/mL
	*E. coli* (ATCC 10536)	62.99 (50–100)	125.99 (100–200)	158.74 (100–200)	39.68 (25–50)	31.49 (25–50)	79.37 (50–100)	125.99 (100–200)	158.74 (100–200)	<0.12
	*P. aeruginosa* (ATCC15442)	125.99 (100–200)	>200	>200	125.99 (100–200)	125.99 (100–200)	>200	>200	125.99 (100–200)	1.23 (0.98–1.95)
	*S. typhi* (ATCC 15442)	>200	125.99 (100–200)	>200	>200	125.99 (100–200)	>200	>200	125.99 (100–200)	0.38 (0.24–0.49)
Bacteria	*Gram+*									
	*B. cereus* (ATCC 12826)	>200	79.37 (50–100)	>200	>200	>200	>200	125.99 (100–200)	>200	<0.12
	*B. subtilis* (PERU 6)	125.99 (100–200)	79.37 (50–100)	125.99 (100–200)	125.99 (100–200)	>200	125.99 (100–200)	>200	>200	<0.12
	*S. aureus.* (ATCC 6538)	62.99 (50–100)	62.99 (50–100)	125.99 (100–200)	>200	79.37 (50–100)	79.37 (50–100)	>200	>200	0.62 (0.98–0.49)
										Griseofulvin, µg/mL
	*T. mentagrophytes* (CCF 4823)	125.99 (100–200)	62.99 (50–100)	125.99 (100–200)	>200	>200	158.74 (100–200)	125.99 (100–200)	>200	2.52 (2–4)
	*T. tonsurans* (CCF 4834)	39.68 (25–50)	39.68 (25–50)	158.74 (100–200)	>200	158.74 (100–200)	125.99 (100–200)	158.74 (100–200)	>200	0.198 (0.125–0.25)
	*T. rubrum* (CCF 4933)	62.99 (50–100)	125.99 (100–200)	62.99 (50–100)	>200	39.68 (25–50)	39.68 (25–50)	39.68 (25–50)	158.74 (100–200)	3.175 (2–4)
	*A. quadrifidum* (CCF 5792)	39.68 (25–50)	62.99 (50–100)	125.99 (100–200)	158.74( 100–200)	79.37 (50–100)	62.99 (50–100)	79.37 (50–100)	125.99 (100–200)	>8
Dermatophytes	*T. erinacei* (CCF 5930)	15.75 (12.5–25)	19.84 (12.5–25)	158.74 (100–200)	>200	>200	>200	>200	>200	3.174 (2–4)
	*A. gypseum* (CCF 6261)	39.68 (25–50)	125.99 (100–200)	125.99 (100–200)	158.74 (100–200)	158.74 (100–200)	125.99 (100–200)	125.99 (100–200)	>200	1.587 (1–2)
	*A. currey* (CCF 5207)	19.84 (12.5–25)	79.37 (50–100)	>200	125.99 (100–200)	>200	39.68 (25–50)	125.99 (100–200)	79.37 (50–100)	>8
	*A. insingulare* (CCF 5417)	31.49 (25–50)	125.99 (100–200)	>200	62.99 (50–100)	>200	125.99 (100–200)	79.37 (50–100)	125.99 (100–200)	>8

a MIC values are reported as geometric means of three independent replicates (*n* = 3). MIC range concentrations are reported within brackets.

In the antifungal assays, *S. tomentosa* demonstrated notable activity against dermatophytes. *Trichophyton erinacei* was the most susceptible species, with MIC values of 12.5–25 µg/mL, whereas *S. heldreichiana* inhibited the same species at 25–50 µg/mL. Similarly, *Arthroderma gypseum*, *A. currey*, and *A. insingulare* showed MIC values one dilution step lower for *S. tomentosa* than for *S. heldreichiana*. Both species exhibited limited activity against yeast strains (*Candida parapsilosis* and *Candida krusei*), with relatively high MIC values.

Recent studies on *S. tomentosa* have confirmed its antimicrobial potential: aqueous extracts of aerial parts exhibited inhibitory activity against *Escherichia coli*, *Listeria monocytogenes*, *Staphylococcus aureus*, and *Candida albicans* with MIC values in the low µg/mL range, indicating substantive inhibitory effects across both bacteria and fungi [88]. In addition, methanolic‐aqueous extracts rich in rosmarinic, salvianolic, and related phenolic acids have been reported to exert significant activity against bacteria causing opportunistic infections, further supporting the broad antimicrobial profile of *S. tomentosa* [20]. By contrast, while *S. heldreichiana* has demonstrated detectable antimicrobial activity in earlier chemical and antimicrobial screenings, recent specific MIC data for this species are still limited. Earlier work on *Salvia* species, including *S. heldreichiana,* showed correlations between phenolic composition and antimicrobial potency, although the activity was generally moderate compared with other *Salvia* taxa.

The reduced susceptibility of Gram‐negative bacteria is consistent with the presence of an outer membrane and intrinsic resistance mechanisms that limit the penetration of plant‐derived compounds, a trend observed across several *Salvia* taxa (e.g., in comparative antimicrobial profiling of *Salvia* hydrolates and extracts against a panel of Gram‐negative and Gram‐positive bacteria [89]. In contrast, the higher sensitivity of Gram‐positive bacteria and dermatophytes suggests that the bioactive constituents of *S. tomentosa* interact more effectively with exposed cell wall and membrane structures.

The superior antimicrobial performance of *S. tomentosa* correlates well with its richer phytochemical profile. LC–MS analysis revealed a higher abundance and diversity of phenolic acids and flavonoids—particularly rosmarinic, caffeic, salvianolic acid derivatives, and luteolin glycosides—which are known to exert antimicrobial effects through membrane disruption, metal chelation, and enzyme inhibition. These compounds are increasingly recognized in recent phytochemical surveys of *Salvia* species for their contribution to antimicrobial efficacy. Although *S. heldreichiana* contains similar classes of metabolites, their lower relative abundance may partly explain its reduced antimicrobial potency.

Overall, these findings support *S. tomentosa* as a promising natural source of antibacterial and antidermatophytic agents. The observed activity, combined with MIC values well below 100 µg/mL in several assays, is in line with recent antimicrobial evaluations of *S. tomentosa* extracts and provides a scientific basis for its traditional use in the treatment of superficial infections ( [88]. Furthermore, these results highlight the potential of *S. tomentosa* for further development through bioassay‐guided fractionation and in vivo validation.

## 4 Conclusion

The current study emphasizes the wide phytochemical diversity and strong bioactivity potential of the two endemic *Salvia* species from Turkish flora, *S. heldreichiana,* and *S. tomentosa*. The extracts from the aerial parts and root samples extracted with various solvents have demonstrated the presence of high amounts of phenolic and flavonoid compounds. Both groups of compounds are well‐accepted antioxidant‐modulating agents and have recognized biological properties. The high antioxidant activities shown in the study, combined with inhibiting activities against acetylcholinesterase, butyrylcholinesterase, alpha‐amylase, and alpha‐glucosidase enzymes, can be a clear indicator of *S. heldreichiana* and *S. tomentosa* plants possessing high bioactivity potential in treating neuropathic and metabolic disorders. Thus, based on this data and ongoing studies using this plant's bioactivity potential on applied pharmacological treatments, new‐generation biotherapeutic agents from *S. heldreichiana* and *S. tomentosa* plants can be developed.

## Author Contributions

**Gokhan Zengin:** conceptualization, methodology, software, formal analysis, investigation, data curation, writing – original draft preparation, writing – review and editing, supervision, project administration, and funding acquisition. **Nilofar Nilofar:** Conceptualization, investigation, and writing – original draft preparation. **Alina Kalyniukova:** conceptualization, methodology, investigation, writing – original draft preparation. **Vasil Andruch:** Conceptualization and validation. **Giancarlo Angeles Flores:** methodology, software, investigation, writing – original draft preparation, and visualization. **Paola Angelini:** methodology, writing – review, and editing. **Roberto Venanzoni**: methodology, validation, writing – review, and editing. **Kubra Aslan:** methodology, investigation, and writing – original draft preparation. **Ilhami Gulcin:** validation, resources, writing – review, and editing. **Evren Yildiztugay:** resources, writing – review and editing.

## Funding

The authors have nothing to report.

## Conflicts of Interest

The authors declare no conflicts of interest.

## Supporting information

Supplementary Material

## Data Availability

The data that support the findings of this study are available on request from the corresponding author.
