# Supplementary material for: An Integrated Metabolomics and Bioactivity Approach to Characterize Salvia heldreichiana and Salvia tomentosa Extracts
Source: ChemistryOpen. 2026 Sep 21;15(10):e70302. doi: 10.1002/open.70302 (PMC13594774; doi:10.1002/open.70302)
Supplement: Supplementary file 1 [file OPEN-15-e70302-s001.pdf]

Table S1. Results of LC-MS-QTOF analysis of *Salvia tomentosa* and *Salvia heldreichiana in* negative ionization mode

**Compounds**	**Formula**	***Salvia tomentosa***								***Salvia heldreichiana***								**LC-MS-QTOF characterization of compounds**											
		**Aerial part**				**Roots**				**Aerial part**				**Roots**															
	**Solvent**	**EA**	**E**	**E-W**	**W**	**EA**	**E**	**E-W**	**W**	**EA**	**E**	**E-W**	**W**	**EA**	**E**	**E-W**	**W**	**Mass**	**RT, min**	**ESI**	**m/z**	**Fr.1**	**Fr.2**	**Fr.3**	**Fr.4**	**Fr.5**	**Fr. 6**	**Fr. 7**	**Fr. 8**
Glutamic acid	C5H7NO3	-	√	√	√	√	√	√	√	-	-	-	-	-	-	-	-	129,967	1,21	[M-H]-	128,0353								
Gluconic/galactonic acid	C6H12O7	√	√	√	√	√	√	√	√	√	√	√	√	√	√	√	√	196,0584	1,31	[M-H]-	195,0505	159,03	105,0195	75,0085					
Arabinoic acid	C6H9O7	√	√	√	√	√	√	√	√	√	√	√	√	√	√	√	√	166,0479	1,32	[M-H]-	165,0298	129,0194	75,0085	59,0141					
Hydroacetophenone	C8H8O2	√	√	√	√	√	√	√	√	-	-	-	-	-	-	-	-	136,0373	1,34	[M-H]-	135,0452	89,0399							
Unknown 210.074	C7H6O3	-	-	-	-	-	-	-	-	√	√	√	√	-	√	√	√	210,074	1,39	[M-H]-	137,0246	108,0217	92,0266	81,0347	53,0395				
Citric acid		-	-	-	-	-	-	-	-	√	-	√	√	-	√	√	√	192,0271	1,94	[M-H]-	147,0451	119,05	93,0336	61,9883					
Succinic acid	C4H6O4	√	√	√	√	√	√	√	√	-	-	-	-	-	-	-	-	118,0267	2,29	[M-H]-	117,0195	73,0295							
Maleic acid	C4H4O4	-	√	√	-	-	√	√	√	-	-	-	-	-	-	-	-	116,0111	2,33	[M-H]-	115,0025	71,0135							
Vanillic acid	C8H8O4	√	√	√	√	√	√	√	√	√	√	√	√	√	√	√	√	168,0425	4,19	[M-H]-	167,035	149,0245	123,0453	109,0302	93,0347	77,03999			
Syringic acid	C9H10O5	√	√	√	√	√	√	√	√	√	√	√	√	√	√	√	√	198,0532	4,58	[M-H]-	197,0453	179,0351	152,8946	135,0252	72,9928				
Dimethylgallic acid	C9H6O3	-	-	-	-	-	-	-	-	√	√	√	√	√	√	√	√	198,053	4,59	[M-H]-	161,0243	133,0294	104,027						
Glucosyl-trihydroxyacetophenone	C9H6O3	-	-	-	-	-	-	-	-	√	√	√	√	√	√	√	√	330,0952	5,01	[M-H]-	161,0244	133,0296	105,0343						
Dihydroxybenzoic acid isomer 1	C7H6O4	√	√	√	√	√	√	√	√	√	√	√	√	√	√	√	√	154,063	5,11	[M-H]-	153,0194	109,0296							
Dihydroxybenzoic acid isomer 2	C7H6O5	√	√	√	√	√	√	√	√	√	√	√	√	√	√	√	√	154,0268	5,28	[M-H]-	153,0194	109,0296							
Caffeoyl-hexoside			√	√	√	√	√	√	√	-	-	-	-	-	-	-	-	448,1589	6,49	[M-H]-	447,1515	293,0876	153,0557	123,0452	89,0239	71,0131			
Unknown 360.1062	C9H6O4	-	-	-	-	-	-	-	-	√	√	√	√	√	√	√	√	360,1062	6,56	[M-H]-	177,0195	133,0296	105,0344						
Hydroxybenzoic acid 1	C7H6O3	√	√	√	√	√	√	√	√	√	√	√	√	√	√	√	√	138,0319	6,84	[M-H]-	137,0245	108,9216	92,9266	81,0342					
Methyl vanillate	C9H10O4	√	√	√	√	√	√	√	√	-	-	-	-	-	-	-	-	182,0581	6,86	[M-H]-	181,0504	163,0399	135,0451	119,0501	107,0495	72,9931			
Unknown 208.03		√	√	√	√	√	√	√	√	-	-	-	-	-	-	-	-	208,0372	7,54	[M-H]-	207,03	163,0398	135,0454	109,0299	79,0562				
Hydroxybenzoic acid 2	C7H6O3	-	-	-	-	-	-	-	-	√	√	√	√	√	√	√	√	138,0318	7,91	[M-H]-	191,02	129,0189	111,0087	87,0085	57,0343				
Salicylic acid glucoside	C10H10O4	-	-	-	-	-	-	-	-	√	√	√	√	√	√	√	√	300,0848	7,92	[M-H]-	193,0506	161,0244	134,0375	133,0297	106,0419				
Unknown 432.1634	C6H12O7	-	-	-	-	-	-	-	-	√	√	√	√	√	√	√	√	432,1634	8,26	[M-H]-	195,0515	177,0412	147,029	129,0195	99,0091	75,0085	59,0135		
Hydroxymethylbenzoic acid	C8H8O3	√	√	√	√	√	√	√	√	-	-	-	-	-	-	-	-	152,0475	8,57	[M-H]-	151,0402	136,0167	108,022	91,0186					
Chlorogenic acid	C16H18O9	√	√	√	√	√	√	√	√	√	√	√	√		√	√	√	354,0954	8,63	[M-H]-	353,0883	191,0562	85,0294						
Caffeoyl sucrose	C21H28O14	√	√	√	√	√	√	√	√	-	-	-	-	-	-	-	-	504,1479	8,82	[M-H]-	503,1401	341,0878	179,035	161,0245	135,045				

Unknown 408.1188	C9H10O5	-	-	-	-	-	-	-	-	√	√	√	√	√	√	√	√	408,1188	8,88	[M-H]-	197,0462	179,0352	152,8964	135,0252	107,0502	72,993			
Unknown 418.1474	C11H10O4	-	-	-	-	-	-	-	-	√	√	√	√	√	√	√	√	418,1474	8,87	[M-H]-	205,0509	161,0244	133,0296						
Unknown 524.1746	C11H12O4	-	-	-	-	-	-	-	-	√	√	√	√	√	√	√	√	524,1746	8,89	[M-H]-	207,0664	179,0349	161,0246	135,0453					
Dihydroxycoumarin	C9H6O4	√	√	√	√	√	√	√	√	√	√	√	√	√	√	√	√	178,0268	9,03	[M-H]-	177,0193	149,0245	133,0295	105,0343	81,0341				
Unknown 306.0775		-	√	√	√	-	√	√	√	√	√	√	√	√	√	√	√	306,0775	9,30	[M-H]-	305,0703	225,113	96,9601						
Caffeic acid	C9H8O4	√	√	√	√	√	√	√	√	√	√	√	√	√	√	√	√	180,0426	9,58	[M-H]-	179,0353	135,0454							
Methylbenzoic acid		-	-	-	-	-	-	-	-	√	√	√	√	√	√	√	√	136,0526	9,59	[M-H]-	281,1393	237	83,0501						
Unknown 416.1319	C18H34O3	-	-	-	-	-	-	-	-	√	√	√	√	√	√	√	√	416,1319	9,94	[M-H]-	297,2442	279,2322							
Unknown 388.1731	C12H16O8	-	-	-	-	-	-	-	-	√	√	√	√	√	√	√	√	388,1731	10,01	[M-H]-	299,0775	137,0246	93,0346						
Luteolin-O-glucoside	$C_{21}H_{20}O_{11}$	√	√	√	√	√	√	√	√		√	√	√		√	√	√	448,1581	10,06	[M-H]-	447,1519	401,1457	269,1031	161,0457	101,0241	71,0135			
Unknown 598.2256		√	√	√	√	√	√	√	√	√	√	√	√	√	√	√	√	598,2256	10,27	[M-H]-	597,2199	387,1445	181,0521	89,0252					
Unknown 634.2036		-	-	-	-	-	-	-	-	-	√	√	√	√	√	√	√	634,2036	10,28	[M-H]-	299,0555	284,033	209,1651	183,8177	151,003	130,0427	81,9574		
Unknown 644.2312		-	-	-	-	-	-	-	-	√	√	√	√	√	√	√	√	644,2312	10,28	[M-H]-	311,2234	293,2125	223,1706	183,014	159,9814				
Tuberoic acid glucoside	C18H28O9	√	√	√	√	√	√	√	√	√	√	√	√	√	√	√	√	388,1736	10,40	[M-H]-	387,1667	207,1031	163,1127	89,0243	71,0137	59,0138			
Epi-hydroxyjasmonic acid glucoside	C18H28O9	√	√	√	√	√	√	√	√	-	-	-	-	-	-	-	-	388,1737	10,51	[M-H]-	387,1666	207,1023	163,1115	89,0244					
Unknown 534.1742	C17H14O6	-	-	-	-	-	-	-	-	√	√	√	√	-	√	-	-	534,1742	10,57	[M-H]-	313,0717	197,0453	179,0349	135,0453	105,035	71,0137			
Unknown 518.1632		√	√	√	√	√	√	√	√	-	-	-	-	-	-	-	-	518,1632	10,80	[M-H]-	517,1576	337,0943	295,0821	235,0616	193,051	134,0374	89,024		
Dihydroxyferulic acod	C16H20O10	-	√	√	√	√	√	√	√	-	-	-	-	-	-	-	-	372,1059	11,19	[M-H]-	371,0988	249,0618	231,0504	121,0297	75,0087				
Dihydroxydimethoxyflavone		-	-	-	-	-	-	-	-	√	√	√	√	√	√	√	√	314,0789	11,19	[M-H]-	313,0718	161,0243	151,0396	133,0295					
Tetrahydroxy-trimethoxyflavone	C17H14O6	-	√	√	√	√	√	√	√	√	√	√	√	√	√	√	√	376,0796	11,19	[M-H]-	313	151,0402	133,0294	123,0445					
Dihydroferulic acid O-glucuronide		-	-	-	-	-	-	-	-	√	√	√	√	√	√	√	√	372,1058	11,20	[M-H]-	313,0732	145,443							
Salicyl phenolic glucuronide		-	-	-	-	-	-	-	-	√	√	√	√	√	√	√	√	314,0637	11,28	[M-H]-	315,1954	271,2078							
Luteolin rutinoside	C27H30O15	-	√	√	√	√	√	√	√		√	√	√		√	√	√	594,1594	11,33	[M-H]-	593,152	473,1089	383,0775	353,067					
Jasmonic acid derivate		√	√	√	√	√	√	√	√	-	-	-	-	-	-	-	-	226,1207	11,41	[M-H]-	225,1131	59,0136							
Unknown 226.1204		-	-	-	-	-	-	-	-	√	√	√	√	√	√	√	√	226,1204	11,42	[M-H]-	327,2179	291,1983	237,1416	209,1162	171,1026	112,098	59,0143		
Flavonoid-O-diglycoside 1		√	√	√	√	√	√	√	√	-	-	-	-	-	-	-	-	520,1795	12,02	[M-H]-	519,1729	311,1141	149,0609						
Unknown 538.1113		-	-	-	-	-	-	-	-	√	√	√	√	√	√	√	√	538,1113	12,26	[M-H]-	327,2179	291,1983	237,1416	209,1162	171,1026	112,098	59,0143		
Hydroxyxinnamaldehyde		-	-	-	-	-	-	-	-	√	√	√	√	√	√	√	√	148,0526	12,62	[M-H]-	329,0882	167,0352	152,0118	123,0457	108,0217				
Unknown 659.169		-	-	-	-	-	-	-	-	√	√	√	√	√	√	√	√	659,169	13,27	[M-H]-	329,1756	314,1532	271,0991						

Unknown 632.1512		-	-	-	-	-	-	-	-	√	√	√	√	√	√	√	√	632,1512	13,27	[M-H]-	331,1902	269,1906	187,0245	172,1058	103,4269	59,0139			
Chalconaringenin di-O glucoside		-	-	-	-	-	-	-	-	√	√	√	√	√	√	√	√	596,1747	13,28	[M-H]-	331,1902	269,1906	187,0245	172,1058	103,4269	59,0139			
Myricitrin		-	-	-	-	-	-	-	-	√	√	√	√	√	√	√	-	464,0954	13,73	[M-H]-	331,1971	304,2003							
Salviaflaside		-	-	-	-	-	-	-	-	-	√	√	√	-	√	√	√	522,1372	14,73	[M-H]-	331,1919	299..1657							
Sagerinic acid	C36H32O16	√	√	√	√	√	√	√	√	√	√	√	√	-	-	-	-	720,1701	15,55	[M-H]-	719,161	539,1191	359,0773	341,0664	197,0456	161,0246			
Luteolin glucuronide/galacturonide	C21H18O12	√	√	√	√	√	√	√	√	-	-	-	-	-	-	-	-	462,0802	15,64	[M-H]-	461,0735	285,0411							
Dihydroxy-C-methylflavanone xylosyl xyloside	C20H30O4	-	-	-	-	-	-	-	-	√	√	√	√	√	√	√	√	534,1738	15,69	[M-H]-	333	315,1977							
Unknown 648.1667		-	-	-	-	-	-	-	-	√	√	√	√	√	√	√	-	648,1667	15,70	[M-H]-	333,2076	315,1946	293,7181	260,2481	187,7355	145,5434	110,546	75,4595	
Quercetin dirhamnoside		-	-	-	-	-	-	-	-	√	√	√	√	√	√	√	√	594,1583	15,77	[M-H]-	333,2068	275,1653							
Kaempferol O-glucoside	C21H20O11	√	√	√	√	√	√	√	√	√	√	√	√	√	√	√	-	448,1006	15,82	[M-H]-	447,0838	285,0405							
Salvianolic acid	C36H30O16	√	√	√	√	√	√	√	√	√	√	√	√	√	√	√	√	718,1543	16,04	[M-H]-	717,1466	673,1552	537,1942	519,094	493,1132	295,0615	203,0354	135,0452	
Ferulic acid	C20H28O5	-	-	-	-	-	-	-	-	√	√	√	√	√	√	√	√	194,0581	16,18	[M-H]-	347,1865	303,1971	257,1912						
Rhamnetin glucoside		-	-	-	-	-	-	-	-	√	√	√	√	√	√	√	√	478,1113	16,48	[M-H]-	347,1869	314,1527							
Quercetin glucoside		-	-	-	-	-	-	-	-	√	√	√	√	√	√	√	√	464,0957	16,68	[M-H]-	347,1869	315,1593	284,0558	245,1539	151,1143	111,045			
Rosmarinate	C18H16O8	√	√	√	√	√	√	√	√	√	√	√	√	√	√	√	√	360,0847	16,86	[M-H]-	359,0771	197,0456	161,0244	135,0451	72,99				
Unknown 582.1946		√	√	√	√	√	√	√	√	-	-	-	-	-	-	-	-	582,1946	17,06	[M-H]-	581,1883	355,119	295,0979	179,056	89,0242				
Unknown 673.1856		√	√	√	√	√	√	√	√	-	-	-	-	-	-	-	-	673,1856	17,23	[M-H]-	672,1778	609,1823	61,9882						
Unknown 646.167		√	√	√	√	√	√	√	√	-	-	-	-	-	-	-	-	646,167	17,23	[M-H]-	645,1607	609,1839	301,0722						
Unknown 612.2052		√	√	√	√	√	√	√	√	-	-	-	-	-	-	-	-	612,2052	17,39	[M-H]-	612,2053	565,1939	385,1304	325,1077	179,0566	119,035	89,0243		
Unknown 596.211		-	-	-	-	-	-	-	-	√	√	√	√	√	√	√	√	596,211	17,95	[M-H]-	349,2033	313,1822	229,1616	153,0928	85,0657				
Unknown 742.1515		√	√	√	√	√	√	√	√	√	√	√	√	√	√	√	√	742,1515	18,32	[M-H]-	349,2033	313,1822	229,1616	153,0928	85,0657				
Unknown 458.0519		√	√	√	√	√	√	√	√	-	-	-	-	-	-	-	-	458,0519	18,33	[M-H]-	457,0451	96,9601							
Umbelliferone putatively	C9H6O3	√	√	√	√	√	√	√	√	-	-	-	-	-	-	-	-	162,0318	18,34	[M-H]-	161,0243	133,0297							
Hydroxycoumarin isomer 1		-	-	-	-	-	-	-	-	√	√	√	√	√	√	√	√	162,0318	18,35	[M-H]-	351,2179	307,2279	115,0767						
Unknown 423.0805		-	-	-	-	-	-	-	-	√	√	√	√	√	√	√	√	423,0805	18,40	[M-H]-	351,2182	333,2073	307,228						
Verbacoside	C29H36O15	-	√	√	√	√	√	√	√	√	√	√	√	√	√	√	√	654,0894	18,48	[M-H]-	653,0831	555,1135	493,114	239,0746					
Unknown 556.122		-	-	-	-	-	-	-	-	√	√	√	√	√	√	√	√	556,122	18,50	[M-H]-	359,0779	197,0459	179,0347	161,0245	133,0296	72,9929			
Unknown 578.1034		-	-	-	-	-	-	-	-	√	√	√	√	√	√	√	√	578,1034	18,53	[M-H]-	359,9779	197,0456	179,035	161					
Unknown 608.1736		-	-	-	-	-	-	-	-	√	√	√	√	√	√	√	√	608,1736	19,08	[M-H]-	359,0985	197,0455	182,0218	152,0557	138,0324	123,0092			

Salvianolic acid b isomer 2		-	-	-	-	-	-	-	-	-	√	√	√	√	√	√	√	718,1535	19,09	[M-H]-	365,1972	347,186							
Unknown 550.2049	C16H20O10	√	√	√	√	√	√	√	√	√	√	√	√	√	√	√	√	550,2049	19,60	[M-H]-	371,0983	249,0616	121,0294	99,0087	77,0396				
Unknown 584.247		-	-	-	-	-	-	-	-	√	√	√	√	√	√	√	√	584,247	20,42	[M-H]-	375,0725	313,0716	269,0824	179,035	135,0452	105,0346			
Unknown 374.1941		√	√	√	√	√	√	√	√	-	-	-	-	-	-	-	-	374,1941	20,62	[M-H]-	373,1872	211,1234	193,1233	119,0345	89,024	71,0136	59,0137		
Unknown 538.1113		-	-	-	-	-	-	-	-	√	√	√	-	√	√	-	-	538,1113	20,78	[M-H]-	376,0676	269,0819	161,0242	151,04	133,0295	61,9881			
Benzylsuccinate	C11H12O4	√	√	√	√	√	√	√	√	√	√	√	√	√	√	√	√	208,0738	20,86	[M-H]-	207,0665	179,035	161,0244	135,0452					
Tetrahydroxyflavone glucuronide		√	√	√	√	√	√	√	√	-	-	-	-	-	-	-	-	462,0797	20,93	[M-H]-	461,073	285,0405							
Unknown 496.1945	C20H20O8	-	-	-	-	-	-	-	-	√	√	√	√	√	√	√	√	496,1945	21,61	[M-H]-	387,109	179,0351	135,0451						
Unknown 564.2211		√	√	√	√	√	√	√	√	√	√	√	√	√	√	√	√	564,2211	21,75	[M-H]-	387,1665	225,077	163,113	89,0243	71,0138	59,0138			
Salvanolic acid	C26H22O10	-	√	√	√	√	√	√	√	√	√	√	√	√	√	√	√	494,1213	21,88	[M-H]-	493,1148	359,0777	295,0614	197,0458	161,0246	135,0451			
Unknown 360.0845		-	-	-	-	-	-	-	-	-	-	√	√	-	√	√	√	360,0845	21,89	[M-H]-	397,2267	96,96	79,9571						
Unknown 636.0776		-	-	-	-	-	-	-	-	-	√	√	√	√	√	√	√	636,0776	21,89	[M-H]-	407,1383	343,1819	238,9305	209,0623	144,4931	81,8683			
Quercetin	C15H10O7	√	√	√	√	√	√	√	-	-	-	-	-	-	-	-	-	302,0428	22,10	[M-H]-	301,0357	178,9986	151,0037	121,0294	107,014	93,0342	83,0136		
Unknown 554.2726		-	-	-	-	-	-	-	-	√	√	√	√	√	√	√	√	554,2726	22,13	[M-H]-	415,1259	207,0664	192,0425	177,0193					
Unknown 610.1325		-	-	-	-	-	-	-	-	√	√	√	√	-	√	√	-	610,1325	22,14	[M-H]-	417,1409	209,0827	173,8833	161,0261	104,1009	73,0972			
Unknown 712.4038		√	√	√	√	√	√	√	√	-	-	-	-	-	-	-	-	712,4038	22,31	[M-H]-	711,3964	665,3922	503,338						
Unknown 206.06		√	√	√	√	√	√	√	√	-	-	-	-	-	-	-	-	206,0581	22,46	[M-H]-	205,0509	161,0255	133,0293						
Eugenin		-	-	-	-	-	-	-	-	√	√	√	√	√	√	√	√	206,0581	22,42	[M-H]-	422,0731	197,0451	179,0347	161,0244	135,0455	72,9929			
Taxifolin arabinoside		-	-	-	-	-	-	-	-	√	√	√	√	√	√	√	√	436,1007	22,96	[M-H]-	427,0562	161,0251	133,0293	112,9857	68,9958				
Unknown 476.1321		-	-	-	-	-	-	-	-	-	-	-	√	-	-	-	√	476,1321	23,03	[M-H]-	431,1564	149,0456	119,05	101,0243	89,0244	59,0137			
Luteolin	C15H10O6	√	√	√	√	√	√	√	√	√	√	√	√	√	√	√	√	286,0481	23,09	[M-H]-	285,9409	151,0035	133,0296	65,0031					
Hesperetin	C16H14O6	√	√	√	√	√	√	√	√	-	-	-	-	-	-	-	-	302,0791	23,15	[M-H]-	301,1815	283,1709							
Unknown 554.2726 2	C21H20O11	-	-	-	-	-	-	-	-	√	√	√	√	√	√	√	√	554,273	23,16	[M-H]-	447,0938	285,0411							
Unknown 540.2208		-	-	-	-	-	-	-	-	√	√	√	√	√	√	√	√	540,2208	23,23	[M-H]-	447,1081	313,0717	267,0662	205,2101	161,025	135,0453			
Unknown 494.1217		-	√	√	√	√	√	√	√	-	-	-	-	-	-	-	-	494,1217	23,28	[M-H]-	493,1148	359,0777	295,0614	197,0458	161,0246	135,0451			
Unknown 284.1778		√	√	√	√	√	√	√	√	-	-	-	-	-	-	-	-	284,1778	23,34	[M-H]-	283,1707	267,1396	253,1233						
Unknown 302.1883		√	√	√	√	√	√	√	√	-	-	-	-	-	-	-	-	302,1883	23,34	[M-H]-	301,1815	283,1711							
Unknown 466.1842		-	-	-	-	-	-	-	-	√	√	√	√	-	√	√	√	466,1842	23,36	[M-H]-	447,151	293,0877	233,0647	191,0546	153,0557	123,045	89,0243	59,0137	
Hydroxypentamethoxyflavone		-	-	-	-	-	-	-	-	-	√	√	-	√	√	√	-	388,1157	23,49	[M-H]-	457,3255	414,3509	313,2331	244,0767	189,6514	145,015	100,9332	55,3229	

Methyl-coumaroyl derivatives		√	√	√	√	√	√	√	-	-	-	-	-	-	-	-	-	316,0583	23,53	[M-H]-	315,9515	271,0249	255,03	243,0299					
Unknown 736.2577		√	√	√	√	√	√	√	√	-	-	-	-	-	-	-	-	736,2577	23,66	[M-H]-	735,3505	505,1701	175,0403						
Luteolin dimethyl ether		√	√	√	√	√	√	√	√	√	√	√	√	√	√	√	√	314,0793	23,73	[M-H]-	313,0723	161,0248	151,0402	133,0297					
Salvianolic acid b isomer 1	C21H20O12	-	-	-	-	-	-	-	-	√	√	√	√	√	√	√	√	718,1532	23,82	[M-H]-	463,0887	300,0279	271,0249	255,0301	151,0035				
Methylated salvianolic acid		√	√	√	√	-	-	-	-	-	-	-	-	-	-	-	-	583,2688	24,02	[M-H]-	582,2612	462,2036	342,1458	316,167	145,0296	119,0503			
Unknown 510.1157		-	-	-	-	-	-	-	-	√	√	√	√	√	√	√	√	510,1157	24,07	[M-H]-	471,3483	296,2307							
Unknown 726.4315		-	-	-	-	-	-	-	-	√	√	√	-	-	-	-	-	726,4315	24,21	[M-H]-	475,1246	313,0714	161,0246	151,0399	133,0294				
Unknown 352.2252	C22H22O12	-	-	-	-	-	-	-	-	√	√	√	√	√	√	√	√	352,2252	24,38	[M-H]-	477,1042	315,0511	299,0199	271,025					
Unknown 314.0793		-	-	-	-	-	-	-	-	√	√	√	√	√	√	√	√	314,0793	24,43	[M-H]-	487,344	443,3429	342,4548	293,1758	241,4047	203,9182	116,9282		
Hydroxycoumarin isomer 2		-	-	-	-	-	-	-	-	√	√	√	√	√	√	√	√	162,0319	24,43	[M-H]-	487,3433	469,3326	453,3392						
Theaflavic acid		-	-	-	-	-	-	-	-	√	√	√	√	√	√	√	√	428,0721	24,43	[M-H]-	493,1144	359,0772	295,052	197,0452	161,0245				
Unknown 377.0751		-	-	-	-	-	-	-	-	√	√	√	√	√	√	√	√	377,0751	24,43	[M-H]-	487,342	469,329	375,2197	249,1514	164,0551	104,7357	71,0504		
Unknown 334.2144	C26H22O10	-	-	-	-	-	-	-	-	√	√	√	√	√	√	√	√	334,2144	24,46	[M-H]-	493,1142	359,0769	197,0458	161,0244	135,0449	72,9928			
Unknown 568.1575		-	-	-	-	-	-	-	-	√	√	√	√	√	√	√	√	568,1575	24,58	[M-H]-	495,1872	167,035	152,0114	108,0216					
Trihydroxy dimethoxyflavone		√	√	√	√	√	√	√	√	-	-	-	-	-	-	-	-	330,0744	24,66	[M-H]-	329,0667	314,0425	299,0197	285,1497	241,1596	199,0401	163,0036	133,0296	
Unknown 380.2203		-	-	-	-	-	-	-	-	√	√	√	-	-	-	-	-	380,2203	24,76	[M-H]-	501,3217	457,3341	439,3226	423,291					
Unknown 350.2097		-	-	-	-	-	-	-	-	√	√	√	√	√	√	-	-	350,2097	24,81	[M-H]-	503,3353	457,3324	397,3377	388,7044	315,1975	246,5595	211,0621	137,0979	95,0878
Unknown 362.173		√	√	√	-	√	√	-	-	-	-	-	-	-	-	-	-	362,173	24,82	[M-H]-	361,1655	317,1762	299,1654	272,1784	243,1026				
Unknown 318.1835		√	√	√	√	√	√	-	-	-	-	-	-	-	-	-	-	318,1835	24,82	[M-H]-	317,1759								
Unknown 366.2047		-	-	-	-	-	-	-	-	√	√	√	-	-	-	-	-	366,2047	25,04	[M-H]-	509,1098	355,0819	313,0721	267,0659	161,0246	150,323	135,0453	109,0292	
Unknown 282.1469		-	-	-	-	-	-	-	-	√	√	√	√	√	√	√	√	282,1469	25,04	[M-H]-	517,3157	485,2553	247,0968	212,0706	142,0879	75,0091			
Unknown 348.1939		-	-	-	-	-	-	-	-	√	√	√	√	√	√	√	√	348,1939	25,06	[M-H]-	519,3316	501,3196	407,2969						
Unknown 348.194		-	-	-	-	-	-	-	-	√	√	√	√	√	√	√	√	348,194	25,066	[M-H]-	521,1305	359,0781	341,088	179,0351	161,0246	135,0345	119,0349	89,0247	
Unknown 334.2146		-	-	-	-	-	-	-	-	√	√	√	√	√	√	√	√	334,2146	25,20	[M-H]-	533,1672	323	209,0822	179,0354	161,0245	135,0453			
Rosmanol/isorosmanol		√	√	-	√	√	√	-	-	-	-	-	-	-	-	-	-	362,2094	25,24	[M-H]-	361,203	317,2128	302,1885	284,1782	201,0927				
Unknown 330.0742		√	√	√	√	-	-	-	-	-	-	-	-	-	-	-	-	330,0742	25,26	[M-H]-	329,1763	314,1533	258,1262						
Hydroxy-carnosic acid / Dehydrocarnosic acid		√	√	√	√	-	-	-	-	-	-	-	-	-	-	-	-	354,241	25,26	[M-H]-	353,2336	335,2232	217,2122						
Unknown 332.1991		-	-	-	-	-	-	-	-	√	√	√	√	√	√	√	√	332,1991	25,41	[M-H]-	533,1676	371,1143	287,0927	179,0349	134,0376				
Unknown 672.1481		√	√	√	√	√	√	√	√	√	√	√	√	√	√	√	√	672,1481	25,41	[M-H]-	537,1107	493,1133		313,0719	295,0612	203,0347	159,0448	135,0454	109,0295

Unknown 538.1475		-	-	-	-	-	-	-	-	-	√	√	-	-	√	√	-	538,1475	25,55	[M-H]-	537,105	493,1143	359,0771	295,0623	197,0457	161,0243	149,024	135,0452	72,9928
Unknown 334.178		-	-	-	-	-	-	-	-	√	√	√	√	√	√	√	√	334,178	25,56	[M-H]-	537,105	493,1143	197,0457	161,0243	135,0452				
Unknown 348.1938		-	-	-	-	√	√	√	√	-	-	-	-	-	-	-	-	348,1938	25,57	[M-H]-	347,1868	301,1818	233,118	179,0714					
Unknown 332.199		√	√	√	√	√	√	√	√	√	√	√	-	√	√	√	√	332,199	25,67	[M-H]-	331,1919	299,1654							
trihydroxy-fatty acid 2		-	-	-	-	-	-	-	-	√	√	√	√	√	√	√	√	328,1677	25,68	[M-H]-	539,2127	495,1257	361,0826	165,0552	150,0329	61,9886			
Trimethoxyflavone / Methoxy-luteolin derivative	$C_{17}H_{14}O_6$	√	√	√	√	√	√	√	√	-	-	-	-	-	-	-	-	314,0791	25,68	[M-H]-	313,0725	298,0486	283,0255	183,0455	163,0039	135,0086	117,0347	65,0031	
Safficinolide	$C_{20}H_{24}O_5$	√	√	√	√	√	√	√	√	√	√	√	√	√	√	√	-	344,0898	25,71	[M-H]-	343,1555	299,1651	243,1028						
Gibberellin 364		√	√	√	-	√	√	-	-	-	-	-	-	-	-	-	-	364,1528	25,73	[M-H]-	363,1453	319,1822	275,1655	247,1705	175,1129	87,0451			
Unknown 398.1942		-	-	-	-	-	-	-	-	-	-	-	-	-	√	√	√	398,1942	25,75	[M-H]-	553,2658	387,167	369,1552	163,1127	59,0139				
Unknown 504.3453		-	-	-	-	√	√	√	√	√	√	√	√	√	√	√	√	504,3453	25,83	[M-H]-	503,3373	458,2162	387,2907	173,0445	149,0245	61,9882			
Unknown 348.1573		√	√	-	-	-	-	-	-	-	-	-	-	-	-	-	-	348,1573	25,84	[M-H]-	347,1868	301,1818	233,118	179,0714					
Chrysoeriol	$C_{16}H_{12}O_6$	√	√	√	√	√	√	√	-	-	-	-	-	-	-	-	-	316,0582	25,87	[M-H]-	315,0515	287,0588	193,0145	165,0193	121,0202				
Flavonoid-O-diglycoside 2		-	-	-	-	-	-	-	-	√	√	√	√	√	√	√	√	520,3399	25,88	[M-H]-	553,2655	387,1656	207,1031	183,1024	59,0138				
Unknown 582.1737		-	-	-	-	-	-	-	-	-	√	√	-	-	√	√	-	582,1737	25,89	[M-H]-	555,1134	493,1134	401,0876	359,0773	295,061	197,0454	161,0244	135,0452	
Unknown 354.2411		√	√	√	√	-	-	-	-	-	-	-	-	-	-	-	-	354,2411	26,05	[M-H]-	353,2341	335,2223	309,2077	161,1335	84,9741				
Carnosic acid	$C_{20}H_{28}O_4$	√	√	√	√	√	√	√	√	-	-	-	-	-	-	-	-	362,2097	26,09	[M-H]-	361,2026	317,2127	302,1888	285,1866					
Unknown 314.1882		√	√	√	-	√	√	√	√	√	√	√	√	√	√	√	√	314,1882	26,09	[M-H]-	313,0723	298,0482	283,0251	255,0298	227,035	183,0455	163,0042	135,0089	117,0341
Unknown 312.1364		√	√	√	√	√	√	√	√	-	-	-	-	-	-	-	-	312,1364	26,11	[M-H]-	311,1291	283,1374	161,026						
Unknown 582.1736		-	-	-	-	-	-	-	-	-	√	√	-	-	√	√	-	582,1736	26,14	[M-H]-	567,1513	405,1203	357,0623	313,071	221,0456	179,0353	161,0245	135,0449	
Methyl rosmarinate	$C_{18}H_{16}O_{10}$	√	√	√	√	√	√	-	-	-	-	-	-	-	-	-	-	300,0634	26,21	[M-H]-	399,0561	284,0318	133,0289						
trihydroxy-fatty acid 1		-	-	-	-	-	-	-	-	√	√	√	√	√	√	√	√	328,1675	26,25	[M-H]-	577,0966	559,0816	515,0962	423,069	381,0585	335,0535	201,0149	179,0355	151,0399
Unknown 330.2828		√	√	√	√	√	√	√	√	-	-	-	-	-	-	-	-	330,2828	26,31	[M-H]-	339,233	171,1043	139,1123						
Unknown 330.0738		√	√	√	√	√	√	-	-	-	-	-	-	-	-	-	-	330,0738	26,34	[M-H]-	329,2338	299,1645	171,1029	139,1133					
Unknown 316.2041		√	√	√	√	√	√	√	√	-	-	-	-	-	-	-	-	316,2041	26,34	[M-H]-	315,1969								
Unknown 332.1987		-	-	-	-	-	-	-	-	√	√	√	√	√	√	√	√	332,1987	26,35	[M-H]-	581,168	401,124	179,0351	135,0449					
Hydroxy-linolenic acid isomer		-	-	-	-	-	-	-	-	√	√	√	√	√	√	√	√	316,204	26,36	[M-H]-	581,168	401,124	179,0351	135,0449					
Pentamethoxyflavan		√	√	√	√	√	√	√	√	-	-	-	-	-	-	-	-	360,1579	26,42	[M-H]-	359,1502	315,1599	272,1057	216,0424	203,0359				
Unknown 350.2093		-	-	-	-	-	-	-	-	√	√	√	√	-	-	-	-	350,2093	26,56	[M-H]-	583,2401	373,1515	209,0829						
Oxidized fatty acid		-	-	-	-	-	-	-	-	√	√	√	√	√	√	√	√	314,1886	26,65	[M-H]-	593,1505	473,108	383,0767	353,0665					

Unknown 316.2042		√	√	√	√	√	√	√	√	-	-	-	-	-	-	-	-	316,2042	26,69	[M-H]-	315,1966	243,1027							
Unknown 352.2251		√	√	√	√	-	-	-	-	-	-	-	-	-	-	-	-	352,2251	26,76	[M-H]-	351,2181	315,1967	307,2264						
Unknown 334.2146		-	-	-	-	-	-	-	-	-	-	-	√	-	-	-	-	334,2146	26,83	[M-H]-	593,1514	285,0406							
Unknown 352.2253		-	-	-	-	-	-	-	-	√	√	√	√	√	√	√	√	352,2253	27,02	[M-H]-	595,1669	287,0561	315,0036	151,0036	135,0452				
Unknown 352.2251		-	-	-	-	-	-	-	-	√	√	√	√	√	√	√	√	352,2251	27,02	[M-H]-	595,1665	287,056	151,0037	135,0037					
Hydroxyluteolin trimethyl ether		-	-	-	-	-	-	-	-	√	√	√	√	√	√	√	√	344,1627	27,11	[M-H]-	597,2177	387,1434	205,0872	181,0505	161,0463	101,0251	89,0243	59,0139	
Unknown 380.1837		√	√	-	-	√	-	-	-	-	-	-	-	-	-	-	-	380,1837	27,22	[M-H]-	379,1765	247,207							
Unknown 348.1939		-	-	-	-	-	-	-	-	√	√	√	√	√	√	√	√	348,1939	27,25	[M-H]-	607,1675	299,0563	284,0327						
Unknown 336.1938		-	-	-	-	√	√	√	√	-	-	-	-	-	-	-	-	336,1938	27,29	[M-H]-	335,2229	273,1862	235,1592	165,0526	131,9556	59,0139			
Giberrelin 348		-	-	-	-	-	-	-	-	√	√	√	√	√	√	√	√	348,1937	27,49	[M-H]-	609,1825	301,0718							
Unknown 518.3244		-	-	-	-	-	-	-	-	√	√	√	√	√	√	√	√	518,3244	27,49	[M-H]-	628,4243	542,3534	427,4458	315,1962	297,2333	238,3708	163,0608	130,9448	100,9326
Hydroxy-oxo-octadecnoic acid		-	-	-	-	-	-	-	-	√	√	√	√	√	√	√	√	312,2302	27,68	[M-H]-									
Unknown 679.4666		-	-	-	-	-	-	-	-	√	√	√	-	-	-	-	-	679,4666	27,73	[M-H]-	633,1949	597,2192	387,1425	181,051	131,0366	89,0245			
Unknown 690.4106		-	-	-	-	-	-	-	-	√	√	√	√	√	√	-	-	690,4106	27,74	[M-H]-									
Unknown 647.4764		-	-	-	-	-	-	-	-	√	√	√	-	√	√	-	-	647,4764	27,74	[M-H]-	643,2227	387,1441	181,0507	166,0266	119,035	101,0244	89,0242	59,0137	
Unknown 316.2042		√	√	√	√	√	√	√	√	-	-	-	-	-	-	-	-	316,2042	28,09	[M-H]-	315,1974	300,1743	271,1719	229,0877	141,2003				
Unknown 502.3293		-	-	-	-	-	-	-	-	√	√	√	√	√	√	√	√	502,3293	28,17	[M-H]-	653,0814	555,1127	493,1138	359,0756	96,96				
Unknown 380.1838		√	√	√	√	√	√	-	-	-	-	-	-	-	-	-	-	380,1838	28,31	[M-H]-	379,2505	335,1912	138,6048	59,0134					
Corosolic acid	$C_{29}H_{36}O_{15}$	-	-	-	-	-	-	-	-	√	√	√	√	√	√	√	√	472,3191	28,31	[M-H]-	653,0806	493,1148	359,0764	96,95999					
Unknown 332.1988		-	-	-	-	-	-	-	-	√	√	√	√	√	√	√	√	332,1988	28,49	[M-H]-	658,163	595,1678	287,0564	151,0037	135,0451	61,9881			
Unknown 486.3345		-	-	-	-	-	-	-	-	√	√	√	√	√	√	√	√	486,3345	28,53	[M-H]-	671,1404	509,1103	359,0793	311,0543	267,0694	197,0457	179,0349	161,0242	135,0453
Unknown 650.4167		-	-	-	-	-	-	-	-	√	√	√	√	√	√	√	-	650,4167	28,57	[M-H]-	680,4632	472,5295	351,2203	333,2081	265,1501	179,0363	61,9886		
Unknown 300.209		-	-	-	-	-	-	-	-	√	√	√	√	√	√	√	√	300,209	28,68	[M-H]-	689,4031	643,4069	584,7496	490,2862	359,1771	333,2087	312,3194	225,8482	190,9752
Unknown 456.3233		-	-	-	-	-	-	-	-	√	√	√	√	√	√	√	√	456,3233	28,68	[M-H]-	717,1463	519,094	339,0512	161,0248	135,0452				
Unknown 785.6157		-	-	-	-	-	-	-	-	√	√	√	√	√	√	√	√	785,6157	28,69	[M-H]-	717,1463	519,094	339,0541	321,0415	295,0624	185,0244			
Unknown 629.4646		-	-	-	-	-	-	-	-	√	√	√	√	√	√	-	-	629,4646	28,73	[M-H]-	717,1457	673,1533	537,1037	519,0936	493,1134	475,1034			
Unknown 801.6117		-	-	-	-	-	-	-	-	√	√	√	√	√	√	√	√	801,6117	28,75	[M-H]-	719,1628	359,0784	197,0458	161,0247	135,045				
Unknown 332.1887		√	√	√	√	√	√	-	-	-	-	-	-	-	-	-	-	332,1887	28,78	[M-H]-	330,1831	314,1524	285,1857						
Unknown 634.3537		-	-	-	-	-	-	-	-	-	-	-	-	-	-	-	-	634,3537	28,81	[M-H]-	633,3829	570,3114	173,0807	130,9465					

Unknown 302.1885		√	√	√	√	√	√	-	-	-	-	-	-	-	-	-	-	302,1885	28,9	[M-H]-	301,1817	258,1257	161,6106	115,2963					
Unknown 318.1833		√	√	√	-	-	-	-	-	-	-	-	-	-	-	-	-	318,1833	28,99	[M-H]-	317,176	299,1644	273,1852	247,1337	219,138				
Unknown 488.3497		-	-	-	-	-	-	-	-	√	√	√	√	√	√	√	√	488,3497	29,07	[M-H]-	784,6086	471,3473	470,336	469,3318					
Putative oxidized fatty acid		-	-	-	-	-	-	-	-	√	√	√	√	√	√	√	√	320,2351	29,45	[M-H]-	800,6065	487,3407	376,4756	215,1213	130,9433	100,9333			

EA - ethyl acetate, E – ethanol, E-W – ethanol-water, W-Water, RT – retention time, min; Fr. - Fragments

Table S2. Results of LC-MS-QTOF analysis of *Salvia tomentosa* and *Salvia heldreichiana* in positive ionization mode

Compounds	Formula	*Salvia tomentosa*								*Salvia heldreichiana*								LC-MS-QTOF characterisation of compounds											
		Aerial part				Roots				Aerial part				Roots															
	Solvent	EA	E	E-W	W	EA	E	E-W	W	EA	E	E-W	W	EA	E	E-W	W	Mass	RT, min	ESI	m/z	Fr.1	Fr.2	Fr.3	Fr.4	Fr.5	Fr.6	Fr.7	Fr.8
Glutamic acid	C5H7NO3	-	√	√	-	-	√	√	√	-	-	-	-	-	-	-	-	129,0788	1,54	[M+H]+	130,0502	84,0442							
Amino acid		-	√	√	-	-	√	√	√	-	-	-	-	-	-	-	-	117,0789	1,60	[M+H]+	118,0846	59,0638							
Unknown 157.11		-	√	√	-	-	√	√	√	-	-	-	-	-	-	-	-	157,1102	2,73	[M+H]+	158,1177	70,065							
Unknown 432.13		√	√	√	√	√	√	√	√	-	-	-	-	-	-	-	-	432,1389	5,59	[M+H]+	433,1472								
Unknown 432.16		√	√	√	√	√	√	√	√	√	√	√	√	√	√	√	√	432,1606	7,96	[M+H]+	433,1686								
Unknown 419.17		√	√	√	√	√	√	√	√	-	-	-	-	-	-	-	-	419,1781	10,10	[M+H]+	420,1843	295,1023	223,0969	150,0768	133,0496	115,0395	97,0288		
Unknown 424.13		√	√	√	√	√	√	√	√	-	-	-	-	-	-	-	-	424,1333	10,10	[M+H]+	425,139								
Hydroxyjasmonic acid	C12H18O4	√	√	√	√	√	√	√	√	-	-	-	-	-	-	-	-	226,1205	10,55	[M+H]+	227,1267	209,1187	191,1075	167,1083	149,0968	131,0857	107,0489	85,0647	
Epi-Hydroxyjasmonic acid glucoside	C18H28O9	√	√	√	√	√	√	√	√	-	-	-	-	-	-	-	-	410,1542	10,55	[M+H]+	411,1625	227,1274	209,1166	191,106	167,1066	149,096	131,0853	85,0645	
Luteolin rutinoside	C27H30O15	-	√	√	√	√	√	√	√	√	√	√	√	√	√	√	√	594,1563	11,37	[M+H]+									
Jasmonic acid derivate		√	√	√	√	√	√	√	√	-	-	-	-	-	-	-	-	226,12	11,45	[M+H]+	227,1287	209,1159	163,1116	131,0846	85,0644	71,0485			
Scopoletin	C10H8O4	√	√	√	√	√	√	√	√	-	-	-	-	-	-	-	-	192,0419	11,95	[M+H]+	193,0494	178,0261	165,0546	133,0282	122,0358	105,0335	94,0408		
Unknown 209.10		√	√	√	√	√	√	√	√	-	-	-	-	-	-	-	-	209,1026	12,04	[M+H]+	210,1102	158,4395							
Unknown 187.12		√	√	√	√	√	√	√	√	-	-	-	-	-	-	-	-	187,1206	12,05	[M+H]+	188,1276	152,1061	142,1218	125,0953	107,0851	83,0851			
Unknown 491.19		√	√	√	√	√	√	√	√	-	-	-	-	-	-	-	-	491,1998	12,06	[M+H]+	492,2079	325,1132	289,0924	163,0602	151,0754	145,0497	127,039	85,0283	
Unknown 496.15		√	√	√	√	√	√	√	√	-	-	-	-	-	-	-	-	496,1551	12,06	[M+H]+	497,1611	335,1087							
Unknown 196.10		√	√	√	√	√	√	√	√	-	-	-	-	-	-	-	-	196,1088	12,98	[M+H]+	197,1173	179,1068	161,0957	135,1165	107,0847	91,0542	67,054		
Unknown 354.16		√	√	√	√	√	√	√	√	-	-	-	-	-	-	-	-	354,1652	14,90	[M+H]+	355,1711								
Unknown 735.17		√	√	√	√	√	√	√	√	-	-	-	-	-	-	-	-	735,1795	16,09	[M+H]+	736,1846	521,1084	411,075	323,0551	181,0497	139,0389			
Unknown 558.17		√	√	√	√	√	√	√	√	-	-	-	-	-	-	-	-	558,171	17,10	[M+H]+	559,1788								
Hesperetin	C16H14O6	√	√	√	√	√	√	√	√	-	-	-	-	-	-	-	-	302,0789	17,28	[M+H]+	303,0857	177,0538	153,0176						
Chrysoeriol-O-glucoside	C22H24O10	√	√	√	√	√	√	√	√	-	-	-	-	-	-	-	-	448,1365	17,28	[M+H]+	449,1436	431,1325	303,0853	195,0284	153,0178	129,0544	85,0279		
Luteolin-O-glucoside		√	√	√	√	√	√	√	√	√	√	√	√	√	√	√	√	610,1888	17,28	[M+H]+	611,1957	449,1433	345,0962	303,0858	263,0542	195,0283	153,0179	85,0281	
Unknown 613.23		√	√	√	√	√	√	√	-	-	-	-	-	-	-	-	-	613,2365	17,98	[M+H]+	614,2433	435,1639	281,1016	263,0913	233,0806	193,0658	161,0596	133,0646	
Rosmarinate	C18H16O8	√	√	√	√	√	√	√	-	-	-	-	-	-	-	-	-	360,0849	18,38	[M+H]+	361,0912	163,039	145,0282	135,0439					

Unknown 742.14		√	√	√	√	√	√	√	-	-	-	-	-	-	-	-	-	742,1483	18,39	[M+H]+	743,1517	383,0738	221,0428	185,0209					
Unknown 382.06		√	√	√	√	√	√	√	√	-	-	-	-	-	-	-	-	382,0661	18,41	[M+H]+	383,071	221,0401	203,028	185,0197	163,0381				
Salvianolic acid 1	C18H12O7	-	√	√	√	√	√	√	√	√	√	√	√	√	√	√	√	340,0584	18,52	[M+H]+	341,0663	323,0548	295,0606	267,0652	251,0714	195,0299	177,0189	163,0391	
Salvianolic acid 2	C18H12O7	-	-	√	√	√	√	√	√								√	340,0581	21,91	[M+H]+	341,0663	323,0548	295,0606	267,0652	251,0714	195,0299	177,0189	163,0391	
Unknown 250.15		-	-	-	√	√	√	√	√	-	-	-	-	-	-	-	-	250,1565	21,95	[M+H]+	251,1643	233,1538	151,076						
Unknown 248.14		-	-	-	√	√	√	√	√	-	-	-	-	-	-	-	-	248,1411	23,24	[M+H]+	249,1486	231,1382	213,1274	185,1321					
Unknown 714.33		√	√	√	√	√	√	√	√	-	-	-	-	-	-	-	-	714,3366	23,39	[M+H]+	715,3462	369,1663							
Unknown 300.17		√	√	√	√	√	√	√	√	-	-	-	-	-	-	-	-	300,1716	23,39	[M+H]+	301,1798	283,1694	259,1332	231,1014	219,1017				
Gibberellin	C20H26O5	√	√	√	√	√	√	√	√	-	-	-	-	-	-	-	-	346,1772	23,39	[M+H]+	347,185	301,1799	283,1693	231,1017	177,091	109,1012			
Unknown 248.14		-	-	-	√	√	√	√	√	-	-	-	-	-	-	-	-	248,1409	23,42	[M+H]+	249,1486	231,1382	213,1274	185,1321					
Unknown 753.28		√	√	√	√	√	√	√	√	-	-	-	-	-	-	-	-	753,2826	23,69	[M+H]+	754,292	737,264	569,186	507,1828	339,1066	231,0864	171,0649		
Unknown 300.17		√	√	√	√	√	√	√	√	-	-	-	-	-	-	-	-	300,172	24,48	[M+H]+	301,1798	283,1694	259,1332	231,1014	219,1017				
Rosmamol/Isorosmanol	C20H26O5	√	√	√	√	√	√	√	√	-	-	-	-	-	-	-	-	346,1773	24,48	[M+H]+	347,185	301,1799	283,1693	231,1017	177,091	109,1012			
Unknown 312.13		√	√	√	√	√	√	√	√	-	-	-	-	-	-	-	-	312,1359	24,73	[M+H]+	313,1436	269,1537	253,0862	237,1498	199,0749	171,0803	155,0856	143,0853	
Apigenin	C15H10O5	√	√	√	√	√	√	√	√	-	-	-	-	-	-	-	-	270,0524	24,76	[M+H]+	271,0604	153,0189	119,0491						
Unknown 262.15		√	√	√	√	√	√	√	√	-	-	-	-	-	-	-	-	262,1561	24,80	[M+H]+	263,1636	245,1538	201,1278	175,0761	145,0651				
Unknown 230.13		√	√	√	√	√	√	√	√	-	-	-	-	-	-	-	-	230,1303	24,80	[M+H]+	231,1361	213,1274	201,1267	186,0968	157,0989	145,0643	93,7363		
Safficinolide	C20H24O5	√	√	-	√	√	√	-	-	-	-	-	-	-	-	-	-	344,1621	24,86	[M+H]+	345,1687	317,1734	299,163	271,1678	231,1008	211,1103	193,0493		
Unknown 297.20		√	√	√	√	√	√	√	√	-	-	-	-	-	-	-	-	297,2092	24,90	[M+H]+	298,2165	228,1387	216,139	200,1066	83,0858	55,0541			
Unknown 260.14		-	-	-	√	√	√	√	√	-	-	-	-	-	-	-	-	260,1402	24,96	[M+H]+	261,149	243,1747	198,9401	153,056	109,0651	71,0498			
Unknown 234.16		√	√	√	√	√	√	√	√	-	-	-	-	-	-	-	-	234,1624	25,13	[M+H]+	235,1691	217,159	189,1279	159,1176	133,101	107,0652	67,0541		
Unknown 230.13		√	√	√	√	√	√	√	√	-	-	-	-	-	-	-	-	230,1302	25,31	[M+H]+	231,1382	175,0657	157,0559	129,063	119,075	91,0489			
Eupatolide		√	√	√	√	√	√	√	√	-	-	-	-	-	-	-	-	248,1409	25,31	[M+H]+	249,1479	231,1375	175,0766	119,0871	85,0663				
Carnosic Acid		√	√	√	√	-	-	-	-	-	-	-	-	-	-	-	-	334,2121	25,42	[M+H]+	335,2224	317,2113	275,1661	233,1532	201,128				
Hydroxy-jasmonate derivative		-	-	-	√	√	√	√	√	-	-	-	-	-	-	-	-	202,1346	25,51	[M+H]+	203,1433	153,0712	117,9793	67,0542					
Unknown 264.17		-	-	-	√	√	√	√	√	-	-	-	-	-	-	-	-	264,171	25,51	[M+H]+	265,1726	247,1692	203,143	159,0809					
Rosmanol		√	√	√	√	√	√	-	-	-	-	-	-	-	-	-	-	328,1651	25,67	[M+H]+	329,1735	314,1499	299,1263						
Dihydroxytrimethoxiflavone	C18H17O7	√	√	√	√	√	√	√	√	-	-	-	-	-	-	-	-	345,2497	25,79	[M+H]+	345,0972	330,0726	312,0631	284,0683	269,0447				
Unknown 292.20		√	√	√	√	√	√	√	√	-	-	-	-	-	-	-	-	292,2019	25,79	[M+H]+	293,1734	275,2032	235,1688	201,1648	173,0575	143,1077	109,1007	83,0842	

Unknown 350.20		√	√	√	√	√	√	√	√	√	√	√	√	√	√	√	-	350,2052	25,79	[M+H]+	351,2147	317,2108	263,1299	223,1321	171,1151				
Unknown 316.05		√	√	√	√	√	√	-	-	-	-	-	-	-	-	-	-	316,0579	25,90	[M+H]+	317,1632	271,159	255,1646						
Unknown 316.20		√	√	√	√	√	√	√	√	-	-	-	-	-	-	-	-	316,2037	26,11	[M+H]+	317,2109	299,2009	205,1229	177,0925	163,073	117,0683	99,0797	81,07	
Unknown 314.18		√	√	√	√	√	√	√	√	-	-	-	-	-	-	-	-	314,1873	26,12	[M+H]+	315,1937	297,1833	259,1311	241,1211	199,0741	187,074	171,0789	81,0693	
Unknown 328.16		√	√	√	√	√	√	√	√	-	-	-	-	-	-	-	-	328,1653	26,56	[M+H]+	329,172	273,1866	207,0659	174,9928	98,9833	55,0534			
Unknown 332.19		√	√	√	-	-	√	√	√	-	-	-	-	-	-	-	-	332,1978	26,71	[M+H]+	333,2234	297,2457	279,2322	261,2224	206,1137				
Keto palmitic acid	C16H30O3	√	√	√	√	√	√	√	√	-	-	-	-	-	-	-	-	270,2182	26,73	[M+H]+	271,1688	253,1567	203,1797	159,0804					
Unknown 230.12		√	√	√	√	√	√	√	√	-	-	-	-	-	-	-	-	230,1294	26,74	[M+H]+	231,2516	213,1266	189,1265	147,0809	111,045	83,0856	58,064		
Unknown 350.20		√	√	√	-	-	-	-	-	-	-	-	-	-	-	-	-	350,207	26,84	[M+H]+	351,2156	333,2063	315,1958	287,2003	271,2055	253,1976	191,105	121,0651	
Unknown 314.07		√	√	√	√	√	√	√	√	-	-	-	-	-	-	-	-	314,0773	26,87	[M+H]+	315,1937	297,1833	259,1311	241,1211	199,0741	187,074	171,0789	81,0693	
Hydroxyluteolin tetramethyl ether	C19H18O7	√	√	√	√	√	-	-	-	-	-	-	-	-	-	-	-	358,1763	26,97	[M+H]+	359,1062	341,3027	298,1497	213,1552	140,1036				
Unknown 334.21		√	√	√	√	√	√	-	-	-	-	-	-	-	-	-	-	334,2136	26,97	[M+H]+	335,221	318,2198	302,2248	205,123	178,0992	165,0911	147,1156	212,1031	
Unknown 204.11		√	√	√	√	√	√	√	√	-	-	-	-	-	-	-	-	204,1149	27,04	[M+H]+	205,1223	163,0758	145,065	107,0501					
Hydroxy-methoxyflavone	C16H12O5	√	√	√	√	√	√	√	-	-	-	-	-	-	-	-	-	284,0675	27,07	[M+H]+	285,0761	248,8649	189,1657						
Unknown 304.16		√	√	√	√	√	-	-	-	-	-	-	-	-	-	-	-	304,1648	27,15	[M+H]+	305,172	259,1674	237,111	193,0815	135,1129				
Unknown 376.18		√	√	√	√	√	√	√	√	-	-	-	-	-	-	-	-	376,1873	27,26	[M+H]+	377,196	299,1635	243,1742	107,0853					
Unknown 230.12		-	-	-	√	√	√	√	√	-	-	-	-	-	-	-	-	230,1296	27,39	[M+H]+	231,1126	185,1092	145,048	101,0116					
Unknown 300.20		√	√	√	√	√	-	-	-	-	-	-	-	-	-	-	-	300,2084	27,44	[M+H]+	301,1802	255,1748	203,1444	149,0964	95,084				
Unknown 335.21		√	√	√	-	-	-	-	-	-	-	-	-	-	-	-	-	335,21	27,45	[M+H]+	336,2258	319,189	301,1786						
Unknown 334.21		√	√	√	-	-	-	-	-	-	-	-	-	-	-	-	-	334,2132	27,45	[M+H]+	335,2216	317,2106	289,2165	149,1319					
Unknown 352.22		√	√	√	-	-	-	-	-	-	-	-	-	-	-	-	-	352,2244	27,45	[M+H]+	353,2307	335,2203							
Unknown 486.33		√	√	√	√	√	√	√	√	-	-	-	-	-	-	-	-	486,3399	27,47	[M+H]+	487,3429	441,338	423,3286	287,1634	185,1333	135,1177			
Unknown 364.18		√	√	-	√	√	-	-	-	-	-	-	-	-	-	-	-	364,1879	27,49	[M+H]+	365,1942	365,1942	347,1844	301,1807	253,1555	205,1228	147,1196	83,0858	
Unknown 232.14		-	-	-	√	√	√	√	√	-	-	-	-	-	-	-	-	232,1455	27,51	[M+H]+	233,1532	215,1428	187,1474	159,1176	159,1176	131,0852	81,0701		
Unknown 778.38		√	√	√	√	√	-	-	-	-	-	-	-	-	-	-	-	778,3893	27,60	[M+H]+	779,8524	637,1553	401,1626	277,1655	217,9819	180,0649			
Unknown 316.20		√	√	√	-	-	-	√	√	√	-	√	-	-	-	-	-	316,2042	27,80	[M+H]+	317,2101	299,2019	247,1337	205,1225	179,1084	121,1023			
Safficinolide		√	√	√	√	√	√	√	√	-	-	-	-	-	-	-	-	344,1988	27,80	[M+H]+	345,2048	299,1963							
Unknown 714.37		√	√	√	√	√	√	-	-	-	-	-	-	-	-	-	-	714,3738	27,80	[M+H]+	715,3766	385,1996	353,1724						
Unknown 332.19		√	√	-	√	√	-	-	-	-	-	-	-	-	-	-	-	332,1952	27,95	[M+H]+	333,2039	301,1788	273,1807	217,1212	151,1092	95,0848			

Unknown 397.28		√	√	√	-	-	-	-	-	-	-	-	-	-	-	-	-	397,2819	28,04	[M+H]+	399,1122	303,2002	287,1277	153,1072					
Unknown 320.23		√	√	√	-	-	-	-	-	-	-	-	-	-	-	-	-	320,2346	28,06	[M+H]+	321,2416	268,1738	179,1041	133,0993	109,1033				
Unknown 621.43		√	√	√	√	√	√	√	√	-	-	-	-	-	-	-	-	621,4378	28,27	[M+H]+	622,3348	568,288	345,2064	325,285	278,2486	161,1343			
Unknown 298.08		√	√	√	√	√	√	√	-	-	-	-	-	-	-	-	-	298,0836	28,29	[M+H]+	299,89	283,0464	267,1726	253,2508	229,1179	204,184	191,1075	179,1083	
Unknown 806.42		√	√	√	-	-	-	-	-	-	-	-	-	-	-	-	-	806,42	28,96	[M+H]+	807,42	773,3496	739,3544	415,2093	381,167	285,2218			
Unknown 390.20		√	√	√	√	√	√	-	-	-	-	-	-	-	-	-	-	390,2036	29,24	[M+H]+	391,2466	356,2838	301,1812	183,1164	149,1006	109,1027			
Unknown 511.54		√	-	√	√	√	-	√	-	-	-	-	-	-	-	-	-	511,5454	29,43	[M+H]+	512,5042	283,2629	256,2632	88,0753					
Glucosamine	C6H13NO5	-	-	-	-	-	-	-	-	-	-	√	-	-	-	√	-	179,0792	1,96	[M+H]+	180,0863	145,0493	127,039	97,0279	85,0283	69,0333			
Unknown 465.1844		-	-	-	-	-	-	-	-	√	√	√	√	√	√	√	√	465,1844	6,54	[M+H]+	466,1923	334,1464	287,1133	251,0916	233,0805	197,0818	179,0704	137,0598	115,039
Unknown 551.2215		-	-	-	-	-	-	-	-	√	√	√	√	-	√	√	√	551,2215	8,26	[M+H]+	552	193,086	161,0599	145,0502					
Phenolic glycoside		-	-	-	-	-	-	-	-	√	√	√	√	-	√	√	√	556,1766	8,26	[M+H]+	557,1842								
Chlorogenic acid	C16H18O9	-	-	-	-	-	-	-	-	√	√	√	√	-	√	√	√	354,0939	8,68	[M+H]+	355,1014	163,0383	145,0278	135,0435	1 170 331				
Unknown 521.2098		-	-	-	-	-	-	-	-	√	√	√	√	√	√	√	√	521,2098	8,70	[M+H]+	522,2181	193,0861	161,0599	115,0387					
Unknown 394.1226		-	-	-	-	-	-	-	-	√	√	√	√	√	√	√	√	394,1226	8,91	[M+H]+	395,1297	232,0698	185,0414						
Ethyl-coumarate		-	-	-	-	-	-	-	-	√	√	√	√	√	√	√	√	192,0776	8,91	[M+H]+	193,0854	161,0586	133,0642	115,0525	105,0691	89,0379	77,0379		
Unknown 389.1676		-	-	-	-	-	-	-	-	√	√	√	√	√	√	√	√	389,1676	8,91	[M+H]+	390,174	193,9861	180,087	161,0598	133,0648	105,0698	85,0285		
Unknown 761.3108		-	-	-	-	-	-	-	-	√	√	√	√	-	√	√	√	761,3108	8,91	[M+H]+	762,3145	211,0962	193,0858	180,0866	161,0598				
Methylcoumarin	C10H8O2	-	-	-	-	-	-	-	-	√	√	√	√	√	√	√	√	160,0542	8,91	[M+H]+	161,059	133,0651	115,0543	105,0698	89,0384	77,0383	55,0174		
Unknown 212.104		-	-	-	-	-	-	-	-	√	√	√	√	√	√	√	√	212,1044	9,37	[M+H]+	213,1123	195,1009	167,0702	135,08	107,0489				
Unknown 391.1834		-	-	-	-	-	-	-	-	√	√	√	√	√	√	√	√	391,1834	9,37	[M+H]+	392,1917	195,1019	167,0703	145,0502	107,0492				
Unknown 396.1389		-	-	-	-	-	-	-	-	√	√	√	√	√	√	√	√	396,1389	9,37	[M+H]+	397,1472	235,0941	185,0421						
Benzylsucciate	C11H10O4	-	-	-	-	-	-	-	-	√	√	√	√	-	√	√	√	208,1087	10,04	[M+H]+	209,0801	177,0531	149,0587	131,0485	103,0537	69,033			
Unknown 405.1993		-	-	-	-	-	-	-	-	√	√	√	√	√	√	√	√	405,1993	10,04	[M+H]+	406,2074	209,1171	191,1065	167,07	149,0961	131,0852	107,0854		
Unknown 419.1784		-	-	-	-	-	-	-	-	√	√	√	√	√	√	√	√	419,1784	10,11	[M+H]+	420,1863	295,1015	259,0808	223,0971	150,0765	133,0495	115,039	97,0281	
Unknown 555.2305		-	-	-	-	-	-	-	-	√	√	√	√	√	√	√	√	555,2305	10,19	[M+H]+	556,2351	521,1984	491,1888	4 551 681	345,1315	329,1375	235,0958	164,0917	71,0489
Unknown 400.1521		-	-	-	-	-	-	-	-	√	√	√	√	√	√	√	√	400,1521	10,31	[M+H]+	401,1596	383,1489	351,1215	330,109	291,1024	247,0963	167,07	123,0434	79,0531
Unknown 615.2516		-	-	-	-	-	-	-	-	√	√	√	√	√	√	√	√	615,2516	10,31	[M+H]+	616,2608	563,212	401,1597	330,1113	247,0966	167,0703			
Hydroxyjasmonic acid	C12H18O3	-	-	-	-	-	-	-	-	√	√	√	√	√	√	√	√	226,12	10,56	[M+H]+	227,1204	209,1088	191,0999	179,0998	167,0983	149,0902	131,0793	107,0799	
Unknown 405.1992		-	-	-	-	-	-	-	-	√	√	√	√	√	√	√	√	405,1992	10,56	[M+H]+	406,2067	209,1175	191,107	167,1071	149,0963	131,0854	85,0643		

Caffeic acid	$C_9H_8O_4$	-	-	-	-	-	-	-	-	√	√	√	√	√	√	√	√	180,042	11,32	[M+H]+	181,0503	153,0545	93,0327						
Rosmaricine	$C_{20}H_{27}NO_4$	-	-	-	-	-	-	-	-	√	√	√	√	√	√	-	-	345,1934	11,41	[M+H]+	346,2018	200,1485							
Jasmonic acid derivate	$C_{12}H_{18}O_3$	-	-	-	-	-	-	-	-	√	√	√	√	√	√	√	√	226,1196	11,46	[M+H]+	227,1204	209,1088	191,0999	179,0998	167,0983	149,0902	131,0793	107,0799	
Unknown 764.2489		-	-	-	-	-	-	-	-	√	√	√	√	√	√	√	√	764,2489	12,61	[M+H]+	765,2523								
Unknown 596.1735		-	-	-	-	-	-	-	-	√	√	√	√	√	√	√	√	596,1735	13,32	[M+H]+	597,1816	435,1296	289,071	195,0292	129,0549	85,0286			
Unknown 556.1543		-	-	-	-	-	-	-	-	√	√	√	√	√	√	√	√	556,1543	15,75	[M+H]+	557	232,0702							
Unknown 735.1777		-	-	-	-	-	-	-	-	√	√	√	√	√	√	√	√	735,1777	16,11	[M+H]+	736,1852	521,1079	411,0711	323,0551	249,0543	181,0492			
Quercetin galactoside/glucoside	$C_{21}H_{20}O_{12}$	-	-	-	-	-	-	-	-	√	√	√	√	√	√	√	√	464,0949	16,75	[M+H]+	465,1019	303,0502	85,0281						
Hydroxycoumarin isomer 1	$C_9H_6O_3$	-	-	-	-	-	-	-	-	√	√	√	√	√	√	√	√	162,0314	18,40	[M+H]+	163,0389	145,0278	135,0434	117,033	89,038				
Myricetin trimethyl ether/Rosmarinate		-	-	-	-	-	-	-	-	√	√	√	√	√	√	√	√	360,084	18,40	[M+H]+	361	163,0392	145,0286	135,0442	1 170 339				
Unknown 737.1942		-	-	-	-	-	-	-	-	√	√	√	√	√	√	√	√	737,1942	18,40	[M+H]+	738,2007	561,1357	441,0713	321,1302	163,0375	105,5763			
Unknown 742.1496		-	-	-	-	-	-	-	-	√	√	√	√	√	√	√	√	742,1496	18,41	[M+H]+	743,1572	383,0721	185,0202						
Unknown 377.1104		-	-	-	-	-	-	-	-	√	√	√	√	√	√	√	√	377,1104	18,41	[M+H]+	378,1176	163,9392	145,0287	135,0441	117,0335				
Unknown 573.1476		-	-	-	-	-	-	-	-	√	√	√	√	√	√	√	√	573,1476	18,55	[M+H]+	574,1562	359,0762	341,0659	181,0496					
Unknown 555.2671		-	-	-	-	-	-	-	-	√	√	√	√	√	√	√	√	555,2671	20,46	[M+H]+	556,2759	311,1486	193,086	1 671 067	149,0961	121,1012	81,0697		
Unknown 560.2225		-	-	-	-	-	-	-	-	√	√	√	√	√	√	√	√	560,2225	20,46	[M+H]+	561,2113								
Unknown 280.1672		-	-	-	-	-	-	-	-	-	-	-	-	√	√	√	√	280,1672	20,61	[M+H]+	281,1739	263,1646	231,1384	203,1437	185,1334	161,132	133,1011		
Unknown 244.109		-	-	-	-	-	-	-	-	-	-	√	√	√	√	√	√	244,109	21,37	[M+H]+	245,1172	217,1224	175,0745	161,0598	147,0806	123,0434	69,0697		
Unknown 282.1827		-	-	-	-	-	-	-	-	-	√	√	√	√	√	√	√	282,1827	22,99	[M+H]+	283,1905	251,1644	221,1528	187,1468	167,1444	125,132	98,1058	67,0527	
Unknown 576.2536		-	-	-	-	-	-	-	-	√	√	√	√	√	√	√	√	576,2536	23,20	[M+H]+	577,258	545,0209	353,0362	295,1541	200,9859				
Unknown 571.2981		-	-	-	-	-	-	-	-	√	√	√	√	√	√	√	√	571,2981	23,20	[M+H]+	572,3026	537,2669	311,1487	227,1274	191,1076	149,0959	121,1009	81,0702	
Unknown 230,13		-	-	-	-	-	-	-	-	-	√	√	√	√	√	√	√	230,1304	23,50	[M+H]+	231,1382	203,1428	175,1113	135,0806	105,0699	91,0546	79,0542	69,0692	
Unknown 530.2469		-	-	-	-	-	-	-	-	-	√	√	√	-	√	-	-	530,2469	24,67	[M+H]+	531,257	309,1311							
Unknown 260.1392		-	-	-	-	-	-	-	-	√	√	√	√	√	√	√	√	260,1392	24,97	[M+H]+	261,1194	243,1127	229,0968	201,1075	161,0803	133,0848	115,0423	77,0298	
Unknown 222.125		-	-	-	-	-	-	-	-	-	-	-	-	√	√	√	√	222,1254	25,04	[M+H]+	223,1331	205,0848	177,1608	161,6238	149,0235	119,0856	105,0707		
Unknown 260.1438		-	-	-	-	-	-	-	-	-	-	-	-	√	√	√	√	260,1438	25,16	[M+H]+	261,1194	243,1127	229,0968	201,1075	161,0803	133,0848	115,0423	77,0298	
Unknown 356.1938		-	-	-	-	-	-	-	-	√	√	√	-	-	-	-	-	356,1938	25,23	[M+H]+	357,2039	339,1886	304,1994	227,1815	143,7097	123,0789	105,0696	75,0261	
Unknown 690.4058		-	-	-	-	-	-	-	-	√	√	√	-	-	-	-	-	690,4058	25,23	[M+H]+	691,4188	515,2354	393,164	357,2024	292,2271	195,929	163,0415		
Unknown 304.1634		-	-	-	-	-	-	-	-	-	-	-	-	√	√	√	√	304,1634	25,31	[M+H]+	303,1726	241,1933	214,8243	133,1014					

Unknown 232.145		-	-	-	-	-	-	-	-	-	-	-	√	√	√	√	√	232,1451	25,31	[M+H]+	233,1538	215,1431	159,0804	109,1009	95,0492	83,0861	67,0541		
Unknown 262.1544		-	-	-	-	-	-	-	-	√	√	√	√	√	√	√	√	262,1544	25,55	[M+H]+	263,1639	231,1382	207,1017	175,148	135,0821	75,0441			
Unknown 262.1556		-	-	-	-	-	-	-	-	√	√	√	√	√	√	√	√	262,1556	25,55	[M+H]+	263,1162	230,1275	203,1412	160,894	142,0757	109,0634	95,0856		
Unknown 500.3111		-	-	-	-	-	-	-	-	-	-	-	-	√	√	√	√	500,3111	25,62	[M+H]+	501,3258	456,0609	417,2748	295,0573	145,0993	107,0849			
Unknown 264.1709		-	-	-	-	-	-	-	-	√	√	√	√	√	√	√	√	264,1709	25,63	[M+H]+	265,1436	247,1342	237,1482	219,1393	205,1598	165,0912	147,117		
Hydroxyluteolin trimethyl ether	C18H16O7	-	-	-	-	-	-	-	-	√	√	√	√	√	√	√	√	344,0881	25,76	[M+H]+	345,2049	275,2015	195,139	155,1068	91,0544		69,0691		
Unknown 274.1927		-	-	-	-	-	-	-	-	√	√	√	√	√	√	√	√	274,1927	25,85	[M+H]+	274,2007	229,1959	173,1338	147,1177	105,0691	91,0547			
Unknown 276.1366		-	-	-	-	-	-	-	-	-	-	-	-	√	√	√	√	276,1366	25,86	[M+H]+	275,1537	215,1345	205,1507	109,0976	95,0458				
Sclareolide	C15H22O2	-	-	-	-	-	-	-	-	-	-	-	-	√	√	√	√	234,1618	26,04	[M+H]+	235,1682	217,1572	173,9212	161,1326	131,083	117,0681	93,0695	85,064	
Unknown 347.1997		-	-	-	-	-	-	-	-	√	√	√	-	-	-	-	-	347,1997	26,16	[M+H]+	348,2121	313,1755	285,1829	239,2347	177,0878	155,1032	121,0645	81,0686	
Hydroxy-linolenic acid isomer	C18H30O3	-	-	-	-	-	-	-	-	√	√	√	-	√	√	√	√	316,2019	26,38	[M+H]+	317,2105	253,1927	189,1313						
Unknown 286.1534		-	-	-	-	-	-	-	-	-	-	-	√	√	√	√	√	286,1534	26,43	[M+H]+	287,1627	217,1238	93,1762						
Unknown 264.1745		-	-	-	-	-	-	-	-	-	√	√	√	√	√	√	√	264,1745	26,43	[M+H]+	265,1798	247,1691	205,1585	147,1171	105,0698	95,9496			
Unknown 246.1609		-	-	-	-	-	-	-	-	-	√	√	√	√	√	√	√	246,1609	26,43	[M+H]+	247,1693	219,1743	204,1514	177,0912	145,1011	119,0864	91,054	67,0542	
Oxidized fatty acid		-	-	-	-	-	-	-	-	√	√	√	√	√	√	√	-	314,0767	26,88	[M+H]+	313,2394	267,1949	169,3337	87,6779	59,0132				
Methyl rosmarinate	C14H18O3	-	-	-	-	-	-	-	-	-	-	√	√	√	√	√	√	230,1298	27,00	[M+H]+	231,1378	213,1248	175,0737	161,0603	143,0863	123,0448	107,0849	91,0546	69,0707
Unknown 369.2497		-	-	-	-	-	-	-	-	√	√	√	-	-	-	-	-	369,2497	27,46	[M+H]+	370,2487	335,2253	283,1545	240,1544	193,1234	147,1187	109,1002		
Unknown 336.1681		-	-	-	-	-	-	-	-	-	-	-	-	√	√	√	√	336,1681	27,69	[M+H]+	335	293,2118	275	265	229	209	183	155	137
Unknown 351.2375		-	-	-	-	-	-	-	-	√	√	√	√	√	√	-	-	351,2375	27,76	[M+H]+	308,2226	291,1944	245,1905	165,0909	112,0761				
Safficinolide	C20H24O5	-	-	-	-	-	-	-	-	-	-	-	-	√	√	√	√	344,161	27,77	[M+H]+									
Unknown 232.145		-	-	-	-	-	-	-	-	-	-	-	-	√	√	√	√	232,1459	27,77	[M+H]+	233,1527	205,0643	197,1315	185,0967	169,0999	159,0798	145,1047	123,0449	105,0699

EA - ethyl acetate, E – ethanol, E-W – ethanol-water, W-Water, RT – retention time, min; Fr. - Fragments
